# Mapping Still Matters: Coarse-Graining with Machine Learning Potentials

**DOI:** 10.1021/acs.jcim.5c03035

**Published:** 2026-02-04

**Authors:** Franz Görlich, Julija Zavadlav

**Affiliations:** † Professorship of Multiscale Modeling of Fluid Materials, Department of Engineering Physics and Computation, TUM School of Engineering and Design, 9184 Technical University of Munich, 80333 Munich, Germany; ‡ Atomistic Modeling Center (AMC), Munich Data Science Institute (MDSI), Technical University of Munich, 85748 Garching, Germany

## Abstract

Coarse-grained (CG) modeling enables molecular simulations to reach time and length scales inaccessible to fully atomistic methods. For classical CG models, the choice of mapping, that is, how atoms are grouped into CG sites, is a major determinant of accuracy and transferability. At the same time, the emergence of machine learning potentials (MLPs) offers new opportunities to build CG models that can in principle learn the true potential of the mean force for any mapping. In this work, we systematically investigate how the choice of mapping influences the representations learned by equivariant MLPs by studying liquid hexane, amino acids, and polyalanine. We find that when the length scales of bonded and nonbonded interactions overlap, unphysical bond permutations can occur. We also demonstrate that correctly encoding species and maintaining stereochemistry are crucial, as neglecting either introduces unphysical symmetries. Our findings provide practical guidance for selecting CG mappings compatible with modern architectures and guide the development of transferable CG models.

## Graphical abstract

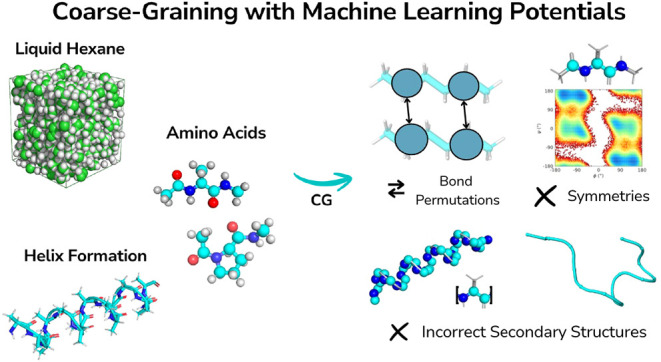

## Introduction

Molecular dynamics (MD) simulations have become an indispensable tool in chemistry, biology, and materials science, offering atomistic insights into the behavior of complex systems. However, a persistent challenge is the vast range of time and length scales governing molecular phenomena. Many processes, such as protein folding or polymer dynamics, occur in microseconds or longer, far exceeding the time scales accessible to conventional all-atom simulations.^1,2^ To bridge this gap, coarse-graining (CG) methods can be used. CG models simplify the system by grouping atoms into fewer interaction sites, or “beads”, thus reducing the effective degrees of freedom. This allows for significantly larger and longer simulations. The central goal of CG models is to preserve the essential structural and thermodynamic properties of the original atomistic system.^2–4^

When developing a CG model, two key questions typically arise: (1) What functional form should be used to represent the CG potential and (2) What mapping scheme should be adopted to define how atoms are grouped into beads? In atomistic systems, only the choice of a potential functional form is relevant, and extensive work has focused on improving its accuracy. In the past decade, attention has been mostly focused on machine learning potentials (MLPs).^5,6^ Their success is based on a paradigm shift that also occurred in other domains, where MLPs learn interactions directly from data, compared to hand-crafted interaction terms of classical potentials.^5,7^

Many modern MLPs incorporate physical symmetries into the model architecture. In particular, E(3)-equivariant neural networks enforce rotational, translational, and reflection symmetries inherent to molecular systems. Architectures such as NequIP,^8^ Allegro^9^ and MACE^10^ that were built on these principles have demonstrated exceptional accuracy and data efficiency. The application of equivariant MLPs to CG modeling is a natural and promising extension.^11–13^ An early study of CG liquid water indicates that equivariant MLPs can drastically reduce the amount of training data needed, performing reasonably well with as little as a single reference frame.^12^ While single-bead mappings are trivial, it is unclear how more general mapping choices affect the learned representation.^12^ In practice, many existing CG-MLPs still rely on priors, i.e., classical energy terms, such as harmonic bond or angle potentials, to maintain molecular connectivity and physical accuracy.^14–18^ This practice reintroduces the manual heuristics that atomistic MLPs were designed to avoid.^19,20^

In this study, we investigate how modern E(3)-equivariant machine learning potentials can be applied to coarse-graining without the use of any priors. Using the MACE architecture as a representative model,^10^ we perform a systematic study to evaluate how the choice of mapping, species encoding and model parameters influence the stability and learned representation of the model. We compare these results with classical CG potentials to assess whether the learned models provide a more accurate description of CG energy landscapes. We evaluated three distinct systems of increasing complexity: liquid hexane, single amino acids, and a 15-mer polyalanine. We validate key findings by additionally testing them with NequIP.^8^

Our results show that the mapping choice can significantly influence the representation that equivariant MLPs learn. In low-resolution mappings of amino acids, we observe symmetries in the free energy surface (FES). We found that these are caused by enantiomerization or ambiguous species encoding. We show that such symmetries propagate from symmetric dihedrals in single amino acids to incorrect secondary structure formation in larger peptides. Finally, we show that bond permutations can occur when bonded and nonbonded length scales overlap, such as in the two-site hexane or *C*_α_ polyalanine model. Together, these findings highlight that while equivariant MLPs offer remarkable flexibility and data efficiency,^12,14^ the preservation of a faithful representation critically depends on the CG mapping.

## Methods

### Mapping in Coarse-Grained Modeling

In CG modeling, the degrees of freedom of an atomistic system are reduced by grouping atoms into beads. Here we limit the study to mappings, in which each atom contributes to at most one CG bead. We use the center-of-mass (COM) mapping to derive CG positions and forces. Other, more general mapping choices,^21,22^ as well as optimal mappings,^23–25^ have also been explored, but remain less prevalent in practice. In the COM mapping, the CG positions $R\in R^{3N}$ are derived as a linear mapping **M** of the atomistic positions $r\in R^{3n}$ , where *N* < *n*. Specifically, the position of CG bead *I* is defined by the weighted sum of the coordinates of the atoms assigned to that bead (eq 1).

1 $$R_{I}=M_{I}\left(r\right)=\sum_{i\in S_{I}}w_{\mathrm{Ii}}r_{i}$$

Here, $S_{I}$ denotes the set of atom indices that contribute to the CG bead *I*. In the COM mapping, the contribution of each atom *w*_*Ii*_ to the CG bead *I* is equivalent to its contribution to the total mass (eq 2).

2 $$w_{I_{i}}=\{\begin{array}{l} \frac{m_{i}}{\sum_{j\in S_{I}}m_{j}},\mathrm{if}\;i\in S_{I}\\ 0,\mathrm{otherwise} \end{array}$$

The weights are normalized such that $\sum_{i\in S_{I}}w_{\mathrm{Ii}}=1$ .^26^ In the COM mapping, the effective force acting on a CG bead f_*I*_ is simply the sum of the atomistic forces^26,27^ (eq 3).

3 $$f_{I}\left(r\right)=\sum_{i\in S_{I}}f_{i}\left(r\right)$$

CG models are parametrized in a “top-down” or “bottom-up” manner. Bottom-up methods^28–30^ use a more detailed reference, for example an atomistic simulation or quantum-mechanics, to derive the CG potential, while top-down approaches^31,32^ aim to reproduce macroscopic observables, such as experimental measurements.^33^ Many models also implement a hybrid approach, for example the MARTINI force field,^34^ which parametrizes nonbonded interactions via experimental partition coefficients, while bonded terms are derived from classical atomistic simulations.^35^ Here we employ force matching, a bottom-up method, to derive the CG potentials. We utilize force matching as it is the standard for training CG-MLPs and directly captures many-body interactions that structure-based methods, like iterative Boltzmann inversion, often miss. Furthermore, force matching avoids the high computational cost of iterative sampling required by strategies such as relative entropy minimization.^15^

### Force Matching

Force matching, first introduced by Izvekov and Voth as the multiscale coarse graining (MS-CG) method,^36^ optimizes the effective force acting on the CG bead f_*I*_ by minimizing the least-squared force residual χ^2^ between the predicted CG and the mapped atomistic forces.^33,37^

$$\chi^{2}\left(\theta \right)=\frac{1}{3N}\left\langle \overset{N}{\underset{I=1}{\sum }}|f_{I}\left(r\right)+\nabla_{I}U_{\mathrm{CG}}\left(M\left(r\right),\theta \right){|}^{2}\right\rangle$$ 4

Here F_*I*_(**R**, θ) = −∇_*I*_ *U*_CG_(**R**, θ) are the predicted forces as a function of the model parameters θ.

### Coarse-Grained Potential

#### Classical Potential

We employ the VOTCA framework^38^ to derive classical CG potentials *U*_Classical_(**R**; θ). The general form is a sum of bonded (bonds, angles, dihedrals) and nonbonded interactions, which are parametrized in the form of splines (eq 5).

5 $$U_{\mathrm{Classical}}\left(R,\theta \right)=\sum_{\mathrm{bond}}U_{\mathrm{bond}}+\sum_{\mathrm{angle}}U_{\mathrm{angle}}+\sum_{\mathrm{dihedral}}U_{\mathrm{dihedrals}}+\sum_{\mathrm{nonbonded}}U_{\mathrm{nonbonded}}$$

We provide a detailed overview of the parametrized interactions and hyperparameters used in the Supporting Information (SI), Table S1.

#### Machine Learning Potential

To parametrize an equivariant MLP *U*_MLP_(**R**; θ), we employ the MACE architecture.^10^ MACE combines the idea of atomic cluster expansion^39^ with message-passing neural networks (MPNNs).^40^ In MACE, the messages passed between nodes, in our case CG beads, are expanded using the idea of a hierarchical body order expansion (eq 6) over neighbors *J*

6 $$m_{I}^{\left(l\right)}=\sum_{J_{1}}u_{1}\left(\sigma_{I}^{\left(l\right)},\sigma_{J_{1}}^{\left(l\right)}\right)+...+\sum_{J_{1},...,J_{\nu }}u_{\nu }\left(\sigma_{I}^{\left(l\right)},\sigma_{J_{1}}^{\left(l\right)},...,\sigma_{J_{\nu }}^{\left(l\right)}\right)$$

where *u*_1...ν_ are learnable functions and σ_*I*_^(*l*)^ is the state of CG bead *I* in layer *l*. The total body order of the model depends on the number of layers *L* and correlation order ν.

We use the chemtrain^41,42^ framework to train the MACE potentials. The training process directly adjusts the model parameters based on eq 4 via backpropagation. A detailed overview of the setup and model hyperparameters can be found in the SI Table.

### Simulations

#### Reference Simulations

Atomistic reference data was generated using GROMACS^43^ in the canonical ensemble (NVT) at 300 K. For liquid hexane, we followed the protocol of Ruehle et al.,^44^ employing the OPLS-AA force field^45^ with a system size of 100 molecules. For peptide systems, we employed the AMBER ff99SB-ILDN force field with the TIP3P water model.^46^ All peptide structures were capped with N-terminal acetyl (ACE) and C-terminal *N*-methyl amide (NME) groups. Production runs were performed for 100 ns (liquid hexane) and 500 ns (peptides). The configurations were sampled uniformly to produce 500,000 training frames.

#### CG Simulations

CG simulations were performed in the NVT ensemble at 300 K. Classical CG simulations were run in GROMACS using stochastic dynamics (SD). MLP simulations were performed using the JAX-MD engine within the chemtrain framework.^41^ A time integration step of 2 fs was used for all CG models. For the implicit solvent baseline of capped amino acids, a time step of 0.5 fs was employed. For capped alanine, we also perform stability tests in a microcanonical (NVE) ensemble. Detailed simulation parameters are provided in the SI.

### Structural Analysis of Coarse-Grained Simulations

#### Radial Distribution Function

To describe nonbonded interactions in the liquid hexane model, we employ radial distribution functions (RDFs). The RDF *g*_*A*–*B*_(*r*) captures the probability of finding a particle of species *A* at a distance *r* from a particle of species *B* (eq 7). It is defined as the ratio of the local density of *A* at a distance *r* from *B* to the bulk density of *A*, ρ_*A*_

7 $$g_{A-B}\left(r\right)=\frac{dN_{A-B}\left(r\right)}{4\pi r^{2}\rho_{A}\mathrm{dr}}$$

where *dN*_*A*–*B*_(*r*) is the average density of *A* particles found in a spherical shell of thickness *dr* at a distance *r* from a central particle *B*, and ρ_*A*_ = *N*_*A*_/*V* is the bulk number density of species *A*. Bonded atoms/CG beads are excluded for the RDF calculation.^47,48^

#### Bonded Parameters

For each liquid hexane mapping, we show the bonded order parameter that embodies the most information: the dihedral angle ϕ_*A*–*B*–*B*–*A*_ for the four-site mapping, the angle θ_*A*–*B*–*A*_ for the three-site mapping, and the bond distance *b*_*A*–A_ for the two-site mapping. To compare the conformational space of single capped amino acids mappings, we evaluated the two backbone dihedrals ϕ_*C*_ACE_–*N*–*C*_α_–*C*_ and ψ_*N*–*C*_α_–*C*–*N*_NME__. We compare 2D representations of the free energy surface (FES) of both dihedrals. The FES was calculated from the joint probability density *P*(ϕ, ψ) using the Boltzmann inversion relation *F*(ϕ, ψ) = −*k*_*B*_ *T* ln *P*(ϕ, ψ).

#### Polyalanine Helix

To analyze the helix formation of the polyalanine peptide, we analyzed different order parameters based on the positions of the *C*_α_ atoms. We adapted the fractional helix content or helicity from Rudzinski et al.^49^ This metric iterates over all *N*_hel_ pairs of *C*_α_ atoms separated by three bonds, corresponding to the hydrogen-bonding pattern charactersitic of α-helices. For each pair, the distance *d*_*ij*_ is computed and scored by how close it is to an optimal distance *d*_0_ = 0.5 nm with variance σ^2^ = 0.02 nm^2^. *Q*_hel_ ranges from 0, completely unstructured (coil or unfolded), to 1, a perfect α helix.

$$Q_{\mathrm{hel}}\left(R=R_{C_{\alpha }}\right)=\frac{1}{N_{\mathrm{hel}}}\underset{i-j=3}{\sum }\exp\left[\left(-1/2\sigma^{2}\right){\left(d_{\mathrm{ij}}-d_{0}\right)}^{2}\right]$$ 8

To differentiate left- from right-handed helices, we used the approach by Sidorova et al.^50^ This method first constructs vectors between neighboring *C*_α_ atoms and then sums the mixed product of consecutive triplets of vectors

9 $$\chi_{\mathrm{hel}}\left(R=R_{C_{\alpha }}\right)=\frac{1}{N_{\mathrm{hel}}}\sum_{i=1}^{N_{C\alpha }-3}v_{i}\cdot \left(v_{i+1}\times v_{i+2}\right)$$

The sign of χ_hel_ determines the handedness or chirality sign of the helix. A positive χ_hel_ indicates a right-handed helix, while negative values indicate a left-handed helix.^50^ The free energy *F*(χ_hel_, *Q*_hel_) was obtained through Boltzmann inversion from the joint probability density. To evaluate the helix propensity of the different mappings, we ranked all reference simulation frames according to |χ_hel_·*Q*_hel_|, selected the 100 frames with the lowest values, and performed 5 ns simulations starting from each configuration.

### Simulation Stability

To assess simulation stability, we applied two different methods. For bulk systems (liquid hexane), we consider the conservation of thermal energy, *k*_B_ *T*. Specifically, we check whether *k*_B_ *T* < 5 kJ/mol (≈2 *k*_B_ *T*), where *k*_B_ is the Boltzmann constant and *T* = 300 K. If a particular frame exceeds this threshold, that frame and all subsequent frames are excluded. We found that the stability classification was insensitive to the exact threshold value within the range of 5–20 kJ/mol (SI, Tables S6 and S7).

For the capped amino acids and polyalanine system, we adapt the distance-based stability criterion of Fu et al.,^51^ by checking whether the distance between the bonded atoms deviates by more than 0.05 nm from the equilibrium bond length. The equilibrium bond length is calculated as the mean of the reference simulation for all matching bond types and frames.

We chose two different strategies, as a distance-based metric is not applicable to all mappings of bulk hexane: Bond distances might change drastically, while thermal energy is preserved (see Results). In the case of polyalanine, the distances might also change due to unphysical bond switching; however, the definition of the polyalanine order parameters depends on the correct order of the *C*_α_ beads. The results of the stability analysis are provided in SI Table S8.

### Well-Tempered Metadynamics

To assess the free energy barrier for enantiomerization, we employ well-tempered metadynamics (WTMetaD).^52^ Unlike standard metadynamics, which builds a bias potential by adding Gaussians of constant height,^53^ WTMetaD ensures convergence by rescaling the Gaussian height according to the accumulated bias (eq 10). The history-dependent bias potential *V*(**s**, *t*) acting on a set of collective variables in the CG space **s**(**R**) is given by

10 $$V\left(s,t\right)=\sum_{t^{\prime }<t}h\;\exp\left(-\frac{V\left(s\left(t^{\prime }\right),t^{\prime }\right)}{k_{B}T\left(\gamma -1\right)}\right)\exp\left(-\sum_{i=1}^{d}\frac{{\left(s_{i}-s_{i}\left(t^{\prime }\right)\right)}^{2}}{2\sigma_{i}^{2}}\right)$$

Here *h* is the initial Gaussian height, σ_*i*_ is the width of the *i*-th collective variable, and γ = T*/*T* is the bias factor. This corresponds to sampling the collective variables at an effective temperature T* = γ*T*.

## Results and Discussion

### Liquid Hexane

We first explore liquid hexane, for which classical CG models have been extensively studied.^44,54–57^ We explore three different mappings: A four-site model, where A-type beads are placed at carbons 2 and 5, and B-type beads are placed at the central carbons 3 and 4.^54^ A three-site model, where the two consecutive carbons and their hydrogens form a bead.^44,55–57^ And finally a two-site model, in which each n-propyl end, or three consecutive carbons and associated hydrogens form a bead.^54,57^ A visual representation can be found in Figure 1. For each mapping, we derive a classical potential and a MLP using MACE.

**Figure 1:**
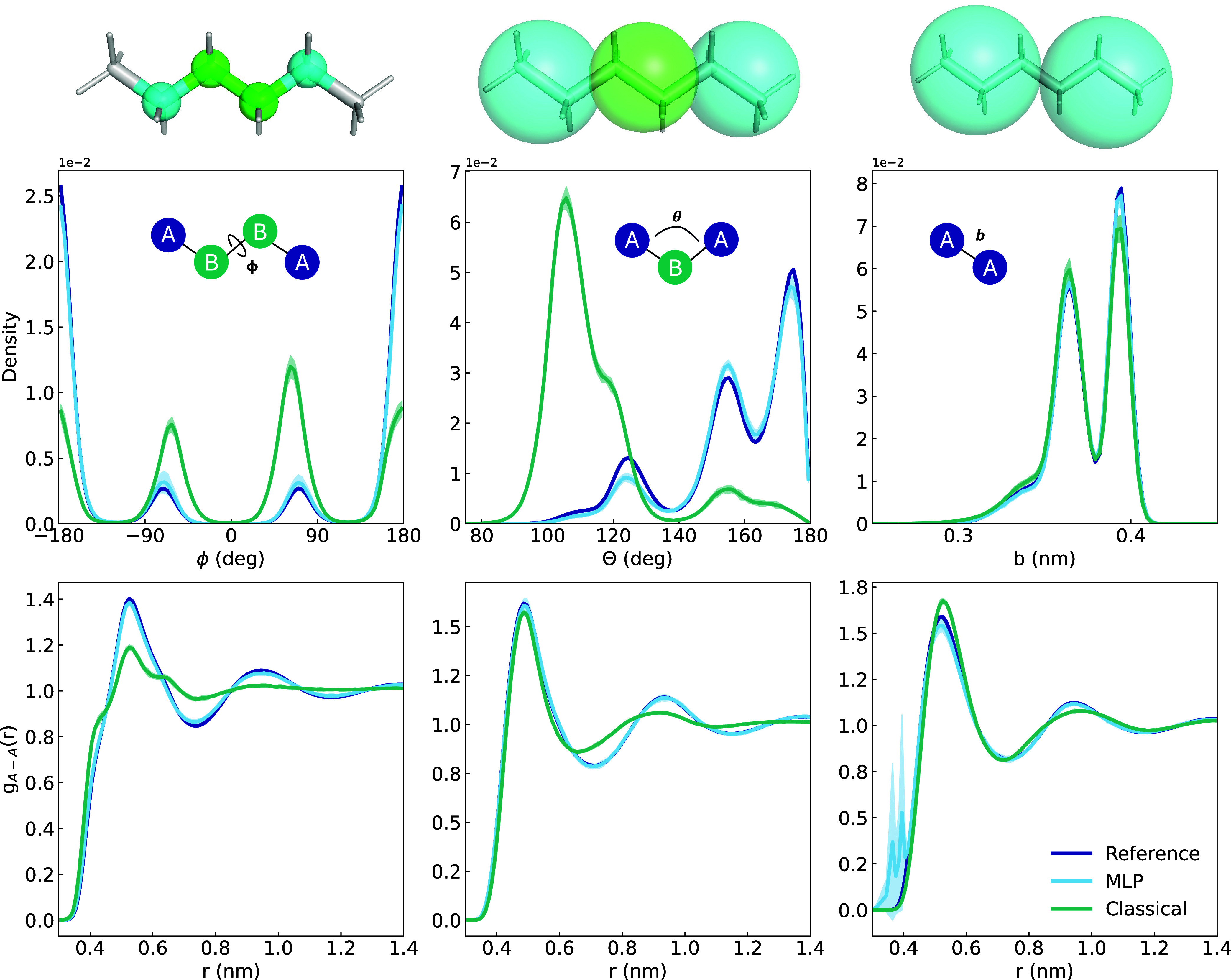
Structural properties of the reference, classical, MLP models for different CG representations of hexane. First row shows bonded population density metrics: Dihedral (four-site model), Angle (three-site model), Bond distance (two-site model). In case of the two-site MLP simulation, we show the nearest neighbor distance instead of bond lengths. The second row shows the RDF of A–A beads. All results show the mean ± 3 standard deviations of 10 × 1000 ps simulations.

#### Four-Site Model

The results for the four-site model can be seen in the first column of Figure 1. Overall, both the classical and MLP are able to replicate the shape of the dihedral population. However, the classical potential overestimates the gauche conformation of hexane with a significant increase in sampling around ± 70 deg. The RDF error is also much smaller for the MLP. We further evaluated the Angular Distribution Functions (ADFs) for A–A–A and B–B–B triplets, which confirm that the MLP accurately captures many-body correlations that are misrepresented by the classical potential (SI, Figure S9).

#### Three-Site Model

For the three-site model, the classical potential fails to sample the A–B–A angle correctly. This mismatch when using force matching with a classical potential has also been observed in other studies of hexane.^38,56^ The reason why force matching fails for the classical potential is that the sum of angle, bond, and nonbonded terms does not consider out-of-plane forces that are produced by a dihedral reorientation in the atomistic model. The functional form can only capture the forces that lie in the plane in which the angle θ is defined.^38^

The MLP is able to closely match the RDF and angular distribution. We tested the effect of the number of message-passing layers *L* and correlation order ν on the ability of the MACE model to reproduce the A–B–A angle correctly (Figure 2a). The total body order of a MACE model is determined by ν and *L* through ∑_*l* = 0_^*L*^ν^*l*^. In the simplest case, *L* = 1 and ν = 1, the total body order is 2 and MACE is unable to capture the angular distribution correctly. Increasing the number of layers or the correlation order allows the MACE model to capture the interaction correctly.

**Figure 2:**
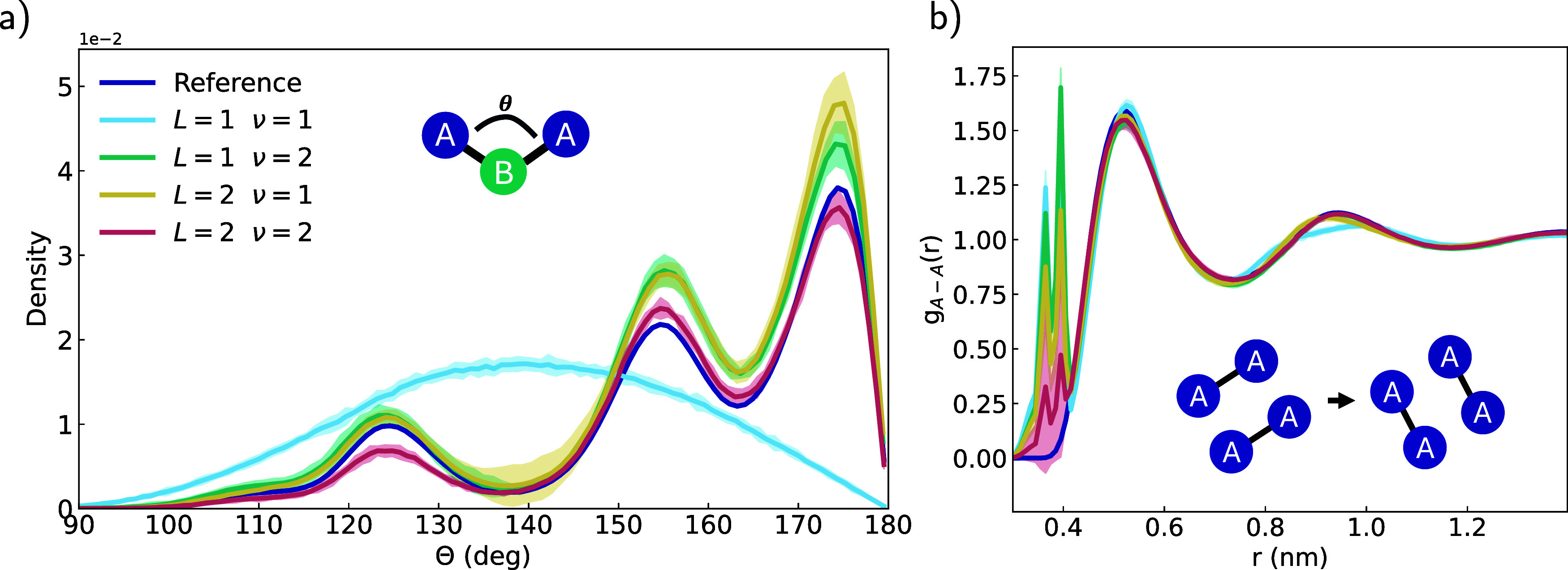
(a) Angular distribution of the hexane three-site model and (b) RDF of the two-site model for different correlation orders ν and number of message-passing layers *L*. In the two-site liquid hexane model, the length scales of bonded and nonbonded interactions overlap. Results show the mean ± 3 standard deviations of 10 × 1000 ps simulations.

#### Two-Site Model

The classical potential performs well in reproducing both bonded and nonbonded interactions of the two-site mapping. For the MLP an interesting behavior was observed: the bond partners switch over the simulation. This means, that an analysis of the bond lengths based on the initial bond list leads to a broadening and diffuse distribution (SI, Figure S1). If instead the closest neighbor of each bead is used for the bond length analysis, the distribution closely matches the reference (Figure 1). The impact of the bond switches is also visible in the RDF, where partners based on the initial bond list are excluded. Two clear peaks are visible at 0.35 and 0.4 nm, an artifact of the newly bonded partners. We tested the influence of ν and *L* on this behavior but could not find any dependence (Figure 2b). We also tested the two-site hexane model with NequIP, observing the same result (SI, Figure S1). While the trends in Figure 2b might suggest that heavier models could alleviate the bond swaps, extended 10 ns simulations using an even heavier model confirm that increased model expressivity only delays the onset of the behavior rather than resolving the underlying mapping ambiguity (SI, Figure S10).

#### Model Parameters

Lastly, we evaluate how the cutoff radius *r*_cut_ and the correlation order ν affect the accuracy, computational performance, and stability of the MACE potential (Table 1). The computational cost of our models is comparable to other CG-MLPs models.^13^ Overall, increasing either the correlation order or the cutoff radius reduces both RDF and force errors. Note that force errors can only be compared between models using the same mapping, as the mapping itself introduces an irreducible noise.^14^ A larger cutoff radius also substantially improves stability, though at the expense of higher computational cost. Similarly, reducing model complexity improves speed; however, classical CG models remain over an order of magnitude faster, achieving around 1600, 2130, and 3470 ns/d for the four-, three-, and two-site models, respectively.

**Table 1:**
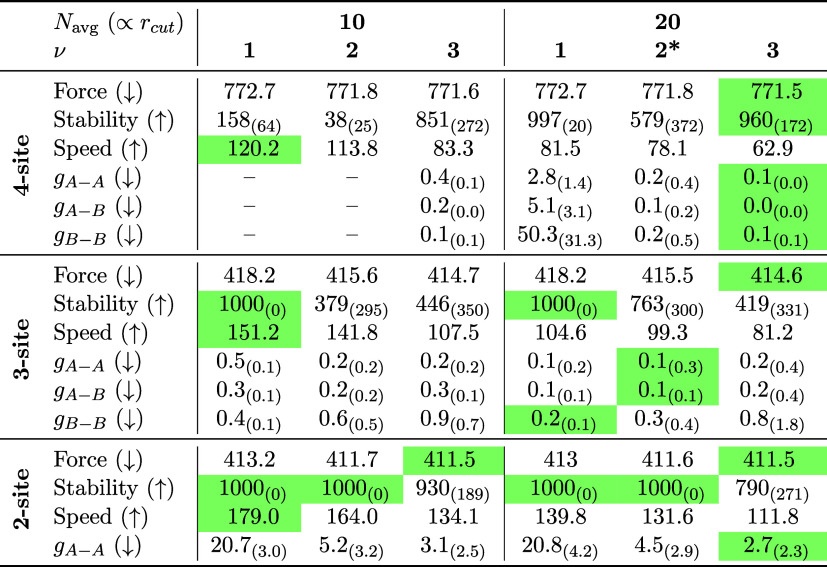
Comparison of a 2-Layer MACE Model with Different Correlation Orders ν and Average Number of Neighbors *N*_avg_ ^a^ ^,^ ^b^

a Results are based on 50 × 1000 ps simulations. Green highlighting indicates better performance (arrows indicate direction: ↑ higher is better, ↓ lower is better). “–” indicates that not enough sample statistics were available. “Force” denotes the Mean Absolute Error (MAE) of the force components evaluated on the validation set (50,000 frames).

b Units: Force in meV/Å, stability in ps, *g*_*A*–A_, *g*_*A*–B_, *g*_*B*–B_ in 10^–3^. Speed is listed in ns/d, based on a single 1000 ps simulation. Subscripts show standard deviations, if applicable. * signifies the model shown in Figure 1.

### Capped Amino Acids

In recent decades, many different CG representations of amino acids have been developed for peptide and protein-related simulations. Mappings can range from near-atomistic representations that only remove hydrogens but preserve heavy-atom movements, to single bead mappings, in which each residue is represented by one bead, for example the *C*_α_-atom.^2^ First, we discuss the widely studied capped l-alanine system (alanine dipeptide). This system has been extensively studied using different machine learning potentials,^14,15,58–60^ as well as more recently generative models.^61,62^ We explore nine different mappings, ranging from an atomistic implicit solvent model to five-bead mappings, which preserve only the central backbone dihedrals. An overview of the mappings can be seen in Figure 3. Fully explicit solvent MLPs were not considered as they can easily get more computationally expensive than the underlying classical atomistic reference, which would negate the efficiency gains sought through CG modeling. Multiscale or adaptive resolution schemes remain potential alternatives for future work.^63–65^ For each mapping, we train a MACE model, and analyze the Ramachandran plot of the backbone dihedrals. We also perform 100 × 100 ps NVE simulations with different time steps to analyze simulation stability (Supporting Information).

**Figure 3:**
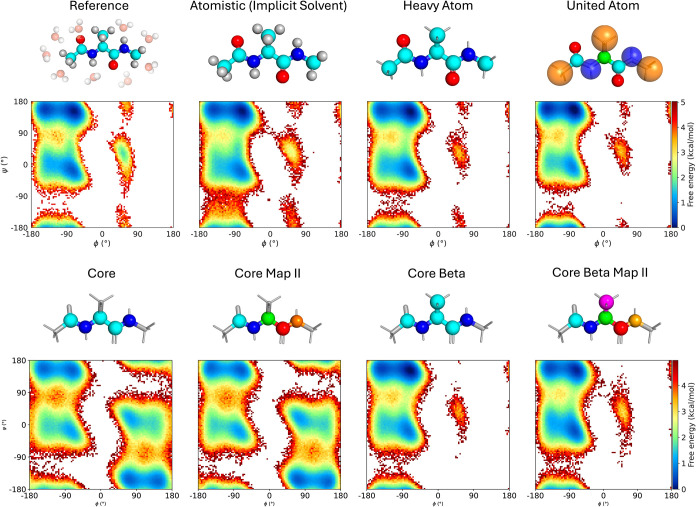
Results of the capped alanine CG simulations with different mappings. The top and bottom rows present the high- and low-resolution mappings, respectively. Each mapping includes a Ramachandran plot derived from 100 × 5 ns simulations.

#### High-Resolution Mappings

Among the high-Resolution mappings considered, the implicit solvent mapping retains all atoms of the capped alanine while removing the solvent molecules that make up the bulk of the system. Further simplification is achieved through Heavy and United Atom mappings, which either drop or merge hydrogens with heavy atoms, respectively. These high-resolution approaches are widely adopted because they preserve essential atomistic dynamics, and therefore allow direct comparisons with fully atomistic models.^13,15^ All high-resolution mappings are capable of accurately modeling the backbone dihedrals (Figure 3). In the NVE simulations, the implicit solvent model remains stable up to time steps of 0.5 fs, while the United and Heavy Atom model can be run safely up to 3 fs.

#### Low-Resolution Mappings

We further investigated low-resolution mappings, which only preserve the atoms essential to the backbone dihedrals, namely the five backbone atoms *C*_ACE_–*N*–*C*_α_–*C*–*N*_NME_ in the “Core” mapping, and additionally the *C*_β_ atom in the “Core Beta” mapping. These mappings are frequently adopted as they define the minimal mapping necessary to capture the essential conformational landscape of the protein backbone.^14,17,59^ For both the Core and Core Beta mapping we test three different species assignments for the CG beads: The atomistic element, a unique species for each bead (Map II), and a single species for all beads (Single).

The Core mappings show a clear point symmetry around the center (Figure 3 column 2). This indicates that the CG molecule during the simulation freely switches between the l- and d-enantiomer. Since both the *C*_α_-hydrogen and the entire side chain are removed, the formal definition of chirality is lost, and a transition between enantiomers becomes an unhindered rotation of the backbone dihedrals. Adding the *C*_β_ atom resolves this issue, and the CG molecule stays in the original l-enantiomer. We observed no noticeable improvement in terms of structural accuracy or stability when using the more detailed species assignment of Map II. A more subtle symmetry was observed when using a single species for all beads. In this case, the model fails to capture the direction of the molecule, which results from an indistinguishability of local environments. We provide these findings, together with evaluations on three other amino acids in the SI Figures S4 and S11.

#### Chiral Inversion

Lastly, we show that CG amino acid models can undergo chiral inversion, i.e., they transition between enantiomers, even if only a single atom is removed around the stereocenter. For that, we calculate the free energy barrier for the transition by biasing the improper *C*_α_-dihedral using WTMetaD. In all cases, we only train on l-alanine. We provide the exact biasing protocol in the SI.

In nature, the direct transition between enantiomers is physically blocked by a high-energy planar transition structure. Consequently, enantiomerization typically requires reactive intermediates.^66,67^ The atomistic (implicit solvent) MLP accurately describes this behavior, and we were unable to observe enantiomerization up to 18 kcal/mol. We find that further increasing the bias factor renders the simulation unstable before any transition can be observed. Classical atomistic force fields would exhibit a similarly insurmountable barrier, as chiral stability is enforced by the combined constraints of harmonic angular potentials or explicit improper dihedral terms.^68^

By removing one atom around the chiral center, in this case the *C*_α_-hydrogen, enantiomerization becomes accessible (Figure 4a). For the Heavy Atom and Core Beta mappings, we find a transition barrier of approximately 13 kcal/mol. The United Atom mapping presents a slightly larger barrier of 16 kcal/mol. This increase arises because merging heavy atoms with their hydrogens shifts the bead COM away from the stereocenter, thereby stabilizing the initial configuration. This also slightly shifts the minima of the improper dihedral.

**Figure 4:**
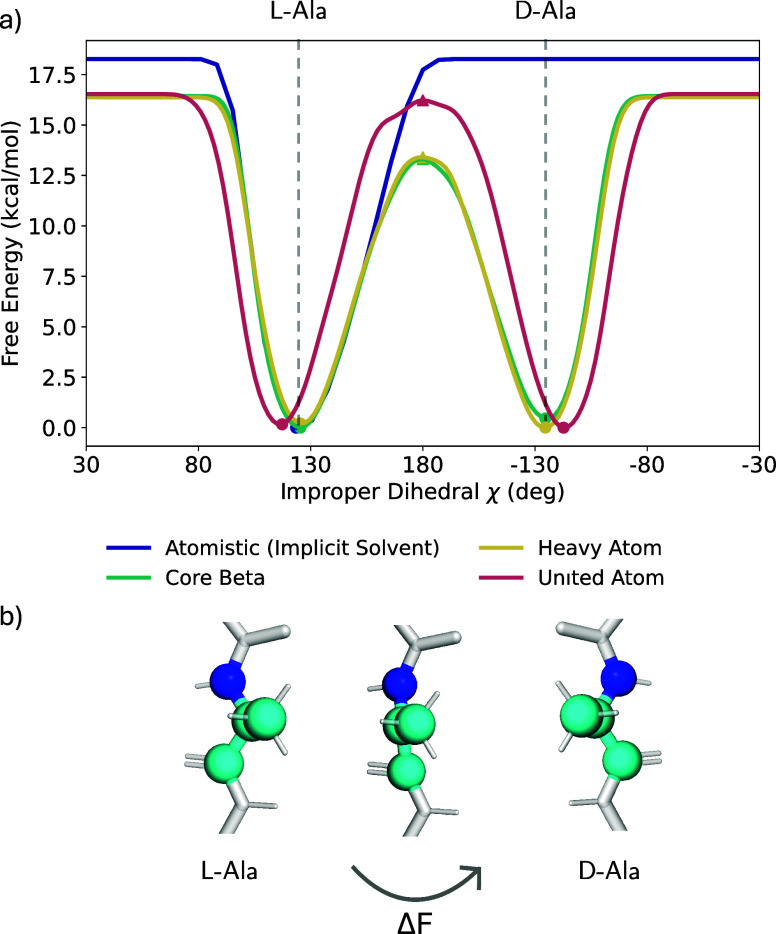
(a) Determined free energy barrier for enantiomerization in atomistic and coarse grained MLPs using WTMetaD along the improper *C*_α_-dihedral. (b) Mechanism of chiral inversion with high-energy planar transition state.

The calculated free energy barriers are very high compared to the thermal energy available of ∼0.6 kcal/mol at 300 K, making transitions very unlikely. At this barrier height, transitions take on the order of micro- to milliseconds. However, increasing the temperature would drastically increase the transition rate, further restricting the state point dependence of the CG potential.

### Helix Formation of Polyalanine

As a last experiment, we extend the low-resolution mappings from capped alanine to a capped 15-mer alanine. Polyalanine systems are good examples of peptides that spontaneously form α helices.^49,69–72^ While naturally occurring L-amino acid peptides form right-handed helices (Figure 5a),^50^ it has been experimentally shown that a d-alanine peptide forms the mirror image, a left-handed helix.^73^

**Figure 5:**
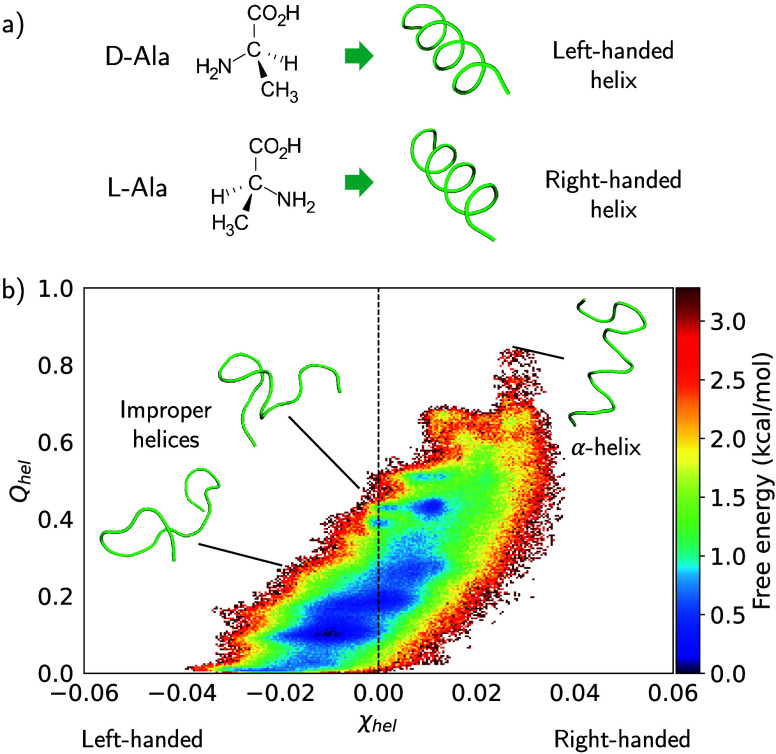
(a) Chain of d-Alanine forms left-handed helices, while the naturally occurring l-Alanine forms right-handed helices. (b) Helicity and handedness of a 500 ns reference simulation of a capped 15-mer l-alanine.

We tested three different mappings: the Core and Core Beta mapping from the capped alanine example, and a *C*_α_ mapping, which only preserves the *C*_α_ atom of each alanine residue. The *C*_α_ mapping is a popular choice for modeling large protein systems.^2,74–76^ In the *C*_α_ mapping, we gave each *C*_α_ a different species. We also performed experiments on three other possible choices: a single species, alternating species, and a symmetric species arrangement. Using unique or alternating species causes unphysical bond switches and ultimately instabilities. We provide the results of all *C*_α_ mappings in the SI Figure S13.

The helix formation of polyalanine can be described via two order parameters: an index of α-helicity *Q*_hel_ (eq 8) and a chirality/handedness index χ_hel_ (eq 9). In the reference atomistic simulation, a clear population of partially folded right-handed α helices can be seen (χ_hel_ > 0 and *Q*_hel_ > 0). Although left-handed helices are visible, these are not proper α-helices (Figure 5b).

When investigating the helicity and handedness of the CG simulations, clear symmetries can be observed (Figure 6). The *C*_α_ mapping is completely symmetric to χ_hel_ = 0, which means that the model does not have a preference for helix handedness. The Core mapping also shows increased sampling of incorrect left-handed helices, even though a preference for the true right-handed helices can be observed. This might be because the starting frames were taken from the l-polylanine reference simulation and are thus favorably oriented to form the right-handed helix. The Core Beta mapping captures the correct helix formation.

**Figure 6:**
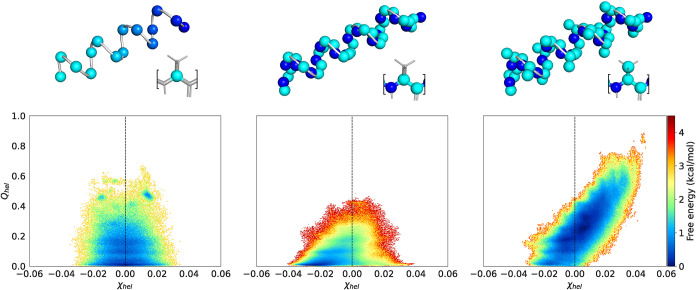
Helicity and handedness of 100 × 5 ns trajectories based on different CG mappings. Left: *C*_α_, where each bead has a unique species embedding. Middle: Core. Right: Core Beta. The top row shows a graphical representation of the ideal helix and per residue mapping.

## Conclusions

Our systematic evaluation of CG models for liquid hexane, amino acids, and polyalanine reveals that the interplay between mapping and model architecture greatly impacts the learned representation of equivariant MLPs. Classical potentials rely on fixed functional forms, which work well for the two-site hexane model but lack the flexibility to capture the more complex many-body correlations present in the three- and four-site liquid hexane models. Although higher-order interactions can in principle be included in classical CG force fields, their number grows combinatorially with interaction order, making both functional form design and parametrization difficult. As a result, most approaches either truncate the expansion at three-body terms or project higher-order many-body effects onto effective pairwise interactions.^77,78^ In contrast, MLPs overcome this barrier by learning many-body interactions directly from data.^17,79^ The MLP performs well for the three- and four-site mappings of hexane, accurately recovering both bonded and nonbonded interactions.

Because MLPs do not include explicit topological information, they cannot distinguish bonded neighbors from nonbonded neighbors. In atomistic systems, these interactions are naturally separable because of their different length scales.^12^ In contrast, for CG representations such as the two-site liquid hexane or polyalanine *C*_α_ model, we showed that these length scales overlap. In these cases, the model fails to distinguish particles, which leads to unphysical bond permutations and ultimately instabilities, in case of the polyalanine *C*_α_ model.

We showed that high-resolution mappings for amino acids can accurately capture atomistic backbone dynamics. However, when further coarsening the representation, we observed spurious symmetries. While equivariant MLPs can distinguish between enantiomers through parity-sensitive features, they will assign the same energy to both mirror images if the output is constrained to a scalar energy. In atomistic systems, this is a correct and desired symmetry, since the transition between enantiomers is correctly modeled with a practically infinite energy barrier. However, we showed that upon removal of the *C*_α_-hydrogen, enantiomerization becomes accessible through a high-energy transition state. If additionally the side chain is removed, the molecule loses its formal chirality and a symmetric FES is obtained. Additionally, bead species have to be chosen carefully, as indistinguishability of local environments can lead to further symmetries.

We confirmed key findings with NequIP, a different E(3)-equivariant MLP.^8^ Our findings likely also extend to invariant graph neural networks, such as SchNet^80^ or DimeNet,^81^ which rely on up to three-body scalar invariants (distances and angles). These models are blind to chirality, making them susceptible to the enantiomerization we observed.^82,83^ Finally, bond permutations are likely to affect any architecture that constructs neighborhoods purely based on geometric information, without topological information.

Overall, equivariant MLPs outperform classical potentials regarding expressivity and data efficiency, theoretically allowing them to learn the potential of mean force for any mapping. In practice, however, our results show that the applicability is limited by mapping-induced artifacts. In scenarios where topological conservation is secondary, such as the liquid hexane systems, MLPs excel at reproducing structural distributions while requiring minimal preparation. However, caution is required when applying MLPs to systems where topology is critical, such as for CG protein models. In these contexts, current MLPs are generally restricted to implicit solvent or Heavy Atom models. Coarser representations, such as the Core and Core Beta mapping, require thought about possible symmetries and overlapping length scales. An even coarser representation, for example the protein *C*_α_-model, risks introducing instabilities, overlapping length scales, or spurious symmetries.

Overall, equivariant MLPs outperform classical potentials regarding expressivity and data efficiency, theoretically allowing them to learn the potential of mean force for any mapping. In practice, however, our results show that the applicability is limited by mapping-induced artifacts. In scenarios where topological conservation is secondary, such as liquid hexane systems, MLPs excel at reproducing structural distributions while requiring minimal preparation. However, caution is required when applying MLPs to systems where topology is critical, such as CG protein models. In these contexts, current MLPs are generally restricted to implicit solvent or Heavy Atom models. Coarser representations, from Core/Core Beta mappings to the *C*_α_-model, risk introducing overlapping length scales or spurious symmetries that lead to simulation instabilities. We confirmed that incorporating a harmonic bond prior eliminates unphysical bond swaps, and similar terms such as excluded volume or improper dihedrals could likely resolve chiral inversion. However, these priors introduce significant trade-offs, as stiff potentials can limit the maximum stable integration time step^17^ and their manual parametrization becomes increasingly difficult as the number of species scales. While priors provide a practical stabilization strategy, our findings highlight the need for architectures that inherently incorporate molecular topology to maintain physical consistency.

## Supplementary Material



## Data Availability

The code and data supporting this study are publicly available at https://github.com/tummfm/CG-Mapping-Benchmark. The training framework chemtrain is publicly available at https://github.com/tummfm/chemtrain.

## Notes

Additional simulation details; classical and machine-learning model parameters; training and stability analysis; theoretical explanation for symmetries; additional analysis on other single capped amino acids; results of other species choices for *C*_α_ polyalanine (PDF)
