# Supplementary material for: Mapping Still Matters: Coarse-Graining with Machine Learning Potentials
Source: J Chem Inf Model. 2026 Feb 4;66(4):2166–76. doi: 10.1021/acs.jcim.5c03035 (PMC12933720; doi:10.1021/acs.jcim.5c03035)
Supplement: Supplementary file 1 [file ci5c03035_si_001.pdf]

Supporting Information
Mapping Still Matters: Learning Coarse-Grained Models with Machine Learning Potentials

Franz Görlich[†] and Julija Zavadlav[*,†,‡]

†*Professorship of Multiscale Modeling of Fluid Materials, Department of Engineering Physics and Computation, TUM School of Engineering and Design, Technical University of Munich, 80333 Munich, Germany*

‡*Atomistic Modeling Center (AMC), Munich Data Science Institute (MDSI), Technical University of Munich, 85748 Garching, Germany*

E-mail: julija.zavadlav@tum.de

General Setup

We used PyMOL Open Source to generate the molecule images.[1]

Data and Software Availability

The code and data supporting this study are publicly available at `https://github.com/tummfm/CG-Mapping-Benchmark`. The training framework `chemtrain` is publicly available at `https://github.com/tummfm/chemtrain`.

Simulation Setup

Atomistic Reference Simulations

Peptides (Explicit Solvent) Simulations were performed using GROMACS with the AMBER ff99SB-ILDN force field and TIP3P water. We used only left-handed (L-)amino acids in all atomistic simulations. The system was prepared by solvating the capped peptide in a cubic box with a minimum distance of 1.2 nm to the box edge. Energy minimization was performed using steepest descent, followed by 100 ps NVT and 100 ps NPT equilibration. Production runs were performed in the NVT ensemble. The temperature was maintained at 300 K using the V-rescale thermostat ($\tau_T = 0.1$ ps) with separate coupling groups for the protein and solvent. Electrostatics were treated using Particle Mesh Ewald (PME) with a fourth-order spline interpolation and a grid spacing of 0.16 nm. A cut-off of 1.0 nm was used for both short-range electrostatic and van der Waals interactions. Equations of motion were integrated using a leap-frog integrator with a time step of 0.5 fs. No bond constraints were applied. The system was simulated for 500 ns and samples were drawn uniformly to yield 500,000 frames.

Liquid Hexane For the liquid hexane system, we follow the protocol of Ruehle et al.,[2] however, we reduce the system size from 1000 to 100 n-hexane molecules and adjusted the box size to obtain the same density. We employed the OPLS-AA force field. An integration step of 1.0 fs was used, with no bond constraints. The system was simulated for 100 ns and samples were drawn uniformly to yield 500,000 frames.

Coarse-grained Simulations

Classical Potential For the CG simulations of liquid hexane, we again follow the protocoll of Ruehle et al.,[2] We perform simulations in GROMACS using the stochastic dynamics (SD) integrator to maintain a temperature of 300 K ($\tau_T = 1.0$ ps) with a time step of 2.0 fs. Non-

bonded interactions were calculated using user-defined tabulated potentials (details below) with a cut-off of 1.3 nm and a table extension of 1.0 nm.

Machine Learning Potential MLP simulations were performed via the JAX MD integration within the `chemtrain` framework.[3,4] The temperature was maintained at 300 K using a Langevin thermostat with a friction coefficient $\gamma = 100.0$ and a time step of 2.0 fs. For the atomistic implicit solvent baseline of capped amino acids, a smaller time step of 0.5 fs was used to ensure stability.

For liquid hexane, we run 50 $\times$ 1000 ps simulations. Since some simulations become unstable, we select 10 fully stable simulations to compare with the classical potential (Figure 1 in main text).

Model Setup

Classical Potential

`VOTCA` offers force matching through their script `csg_fmatch`, which uses splines to describe the different interaction potentials with trainable prefactors θ.[5] The parametrized interactions are listed in table S1. For every force matching step, we used 20,000 frames and the constrained least-squares solver. We used the full 500,000 samples for training the classical potential.

Table S1: Parameters for VOTCA force matching. Min and Max characterise start and end of the force matching range, Bin Size the length of each spline, and Out Step the intervals for the final table.

Interaction	Min	Max	Bin Size	Out step
**Two-site**				
g_{A-A}	0.34	1.0	0.02	0.01
b_{AA}	0.32	0.42	0.005	0.001
**Three-site**				
g_{A-A}	0.34	1.0	0.02	0.01
g_{A-B}	0.34	1.0	0.02	0.01
g_{B-B}	0.34	1.0	0.02	0.01
b_{AA}	0.22	0.28	0.005	0.001
θ_{ABA}	1.6	3.1	0.05	0.01
**Four-site**				
g_{A-A}	0.34	1.0	0.02	0.01
g_{A-B}	0.34	1.0	0.02	0.01
g_{B-B}	0.34	1.0	0.02	0.01
b_{AB}	0.22	0.28	0.005	0.001
θ_{ABA}	1.8	2.2	0.05	0.01
ϕ_{ABBA}	−3.14	3.14	0.314	0.157

The force tables were smoothed and integrated via csg_call to obtain the potential tables. Finally, we used the 2019 version of GROMACS,[6] which still supports bonded potential tables via the -tableb flag to run the CG simulation.

Machine Learning Potentials

We used a JAX implementation of the models and trained the models using chemtrain framework.[4]

Model Parameters - MACE In Table S2 we list the model parameters for MACE that were used, if not stated otherwise.

Table S2: Model parameters that apply to all training runs.

**Parameter**	**Value**
Hidden irreducible representations	32×0e + 32×1o
Readout irreducible representations	16×0e
Output irreducible representations	1×0e
Correlation order	2
Number of message passing layers	2
Maximum angular momentum number	3
Interaction irreducible representations	`o3_restricted`
Number of radial basis functions	8

Model Parameters - NequIP In Table S3 we list the default model parameters for NequIP as defined in the configuration.

Table S3: Model parameters for the NequIP training runs.

**Parameter**	**Value**
Embedding dimension	32
Hidden irreducible representations	16×0e + 16×1o + 16×1e + 2×2e
Input irreducible representations	1×1o
Output irreducible representations	8×0e, 2×0e
Maximum angular momentum	2
Number of message passing layers	4
MLP hidden dimension	64
MLP layers	2
Number of radial basis functions	8

Model Parameters - Cutoff distances In Table S4 we list all explored mappings and the cutoff that was used for training and simulation.

Table S4: Cutoff radius in Angstroms for different N_{avg}. Exact average neighbor counts given in parentheses.

**Model**	N_{avg}	**Cutoff Radius (Å)**
**Liquid Hexane**		
Two-site ($N_{avg} \approx 10$)	10.54	6.5
Two-site ($N_{avg} \approx 20$)	18.2	8.0
Three-site ($N_{avg} \approx 10$)	9.14	5.5
Three-site ($N_{avg} \approx 20$)	19.5	7.0
Four-site ($N_{avg} \approx 10$)	11.2	5.5
Four-site ($N_{avg} \approx 20$)	20.85	6.5
**Peptides**		
Atomistic	–	5.0
Heavy Atom	–	5.0
United Atom	–	5.0
Core	–	5.0
Core Map II	–	5.0
Core Single	–	5.0
Core Beta	–	5.0
Core Beta Map II	–	5.0
Core Beta Single	–	5.0
CA Map I–IV	–	12.0

Training Setup Training was performed using force-matching on the selected datasets. The key hyperparameters and training procedure are summarized in Table S5.

Table S5: Training and optimization hyperparameters.

**Parameter**	**Value**
Optimizer	Adam
Loss Function	Force Matching
Data Split (Train/Val)	90%/10%
Batch Size	32
Initial Learning Rate	1×10^{-3}
Learning Rate Schedule	Exponential Decay
Decay Rate	0.9 (over total steps)
Gradient Clipping	1.0
Epochs	50

Trainings were performed on a single A100 or RTX 3090 GPU using `float32` precision. We used the Adam optimizer with a gradient clipping threshold to prevent exploding gradi-

ents. The learning rate was modulated using an exponential decay schedule, decreasing by a factor of 0.9 over the course of all training steps. The final model was selected based on the lowest validation loss during training.

Supplementary Results

NequIP

In Figure S1 we show the results of the NequIP[7] model on the low-resolution capped alanine and liquid hexane systems. The resulting symmetries and bond permutations are the same as found with MACE (main text).

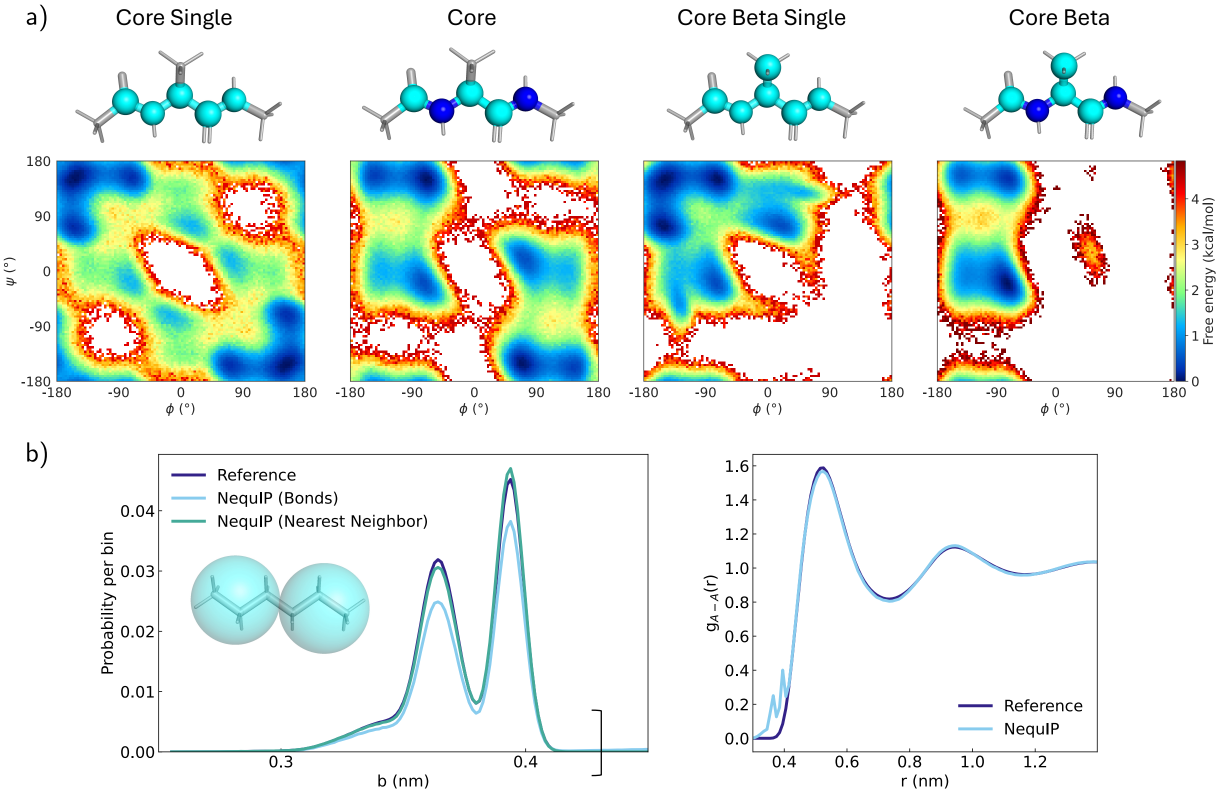

Figure S1: Key findings tested with NequIP. (a) Ramachandran plots of 100 x 5 ns NVT simulations for low resolution mappings of capped alanine. (b) Bond lengths and A-A RDF of 10 x 1 ns NVT simulations for the liquid hexane two-site model. The x-axis of the bond length distribution was truncated at 0.45 nm.

In Figure S1b, we show the bond lengths based on the initial bond partner list, as well as the A-A bead RDF. We truncate the bond length distribution at 0.45 nm, as the NequIP model shows a broad distribution, due to the newly bonded partners. These are also visible in the RDF.

Prior Potential

To investigate the bond swaps we observed in our two-site CG-MLP models of liquid hexane further, we implemented a harmonic bond prior potential and trained a hybrid model. The harmonic prior is defined as:

$$U_{prior}(b_i) = \frac{k_B T}{2\sigma_{b,AT}^2}(b_i - \langle b_i \rangle_{AT})^2 \quad (1)$$

where b_i represents the bond length, $\langle b_i \rangle_{AT}$ is the equilibrium bond distance from the mapped atomistic reference, and $\sigma_{b,AT}^2$ is the variance of the mapped atomistic bond length distribution.

We integrated this prior into a MACE model ($L = 2, \nu = 2$) by combining the energy prediction of the MLP with the harmonic energy prior. As shown in Figure S2, the inclusion of the prior effectively regularizes the model. By explicitly penalizing large deviations from the bonded equilibrium, the model is forced to differentiate between bonded and non-bonded neighbors even when they are at overlapping distances. This successfully eliminated the artifactual peaks in the Radial Distribution Function (RDF) at $\approx$ 0.35 nm, as the bonded partners remained fixed throughout the 50 x 1 ns production runs (Figure S2b).

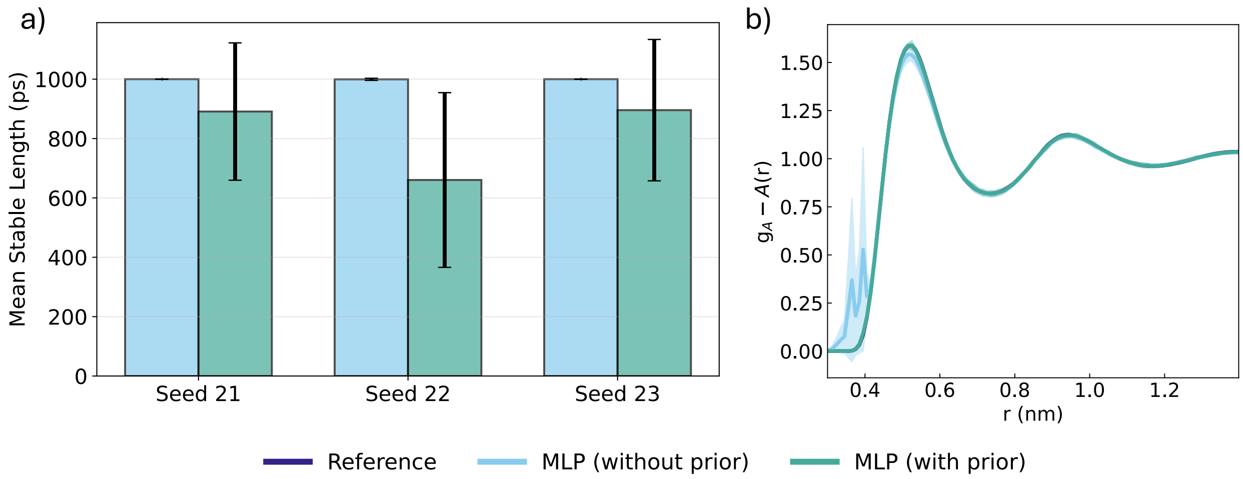

Figure S2: Results of adding an harmonic prior potential to the two-site liquid hexane CG-MLP. (a) Mean and standard deviation of the simulation stability of 50 x 1 ns simulations with the CG-MLP with and without prior. (b) RDF of the first model, showing 10 x 1 ns that remained stable.

However, while the prior potential resolves the topological ambiguity, we observed a notable trade-off in numerical stability. As illustrated in Figure S2a, models trained with the prior showed a higher tendency to crash or deviate from stable integration compared to the pure MLP versions. This suggests that while a simple harmonic prior can enforce structural constraints, it may introduce stiffness or force inconsistencies that the GNN struggles to smooth out.

Symmetries

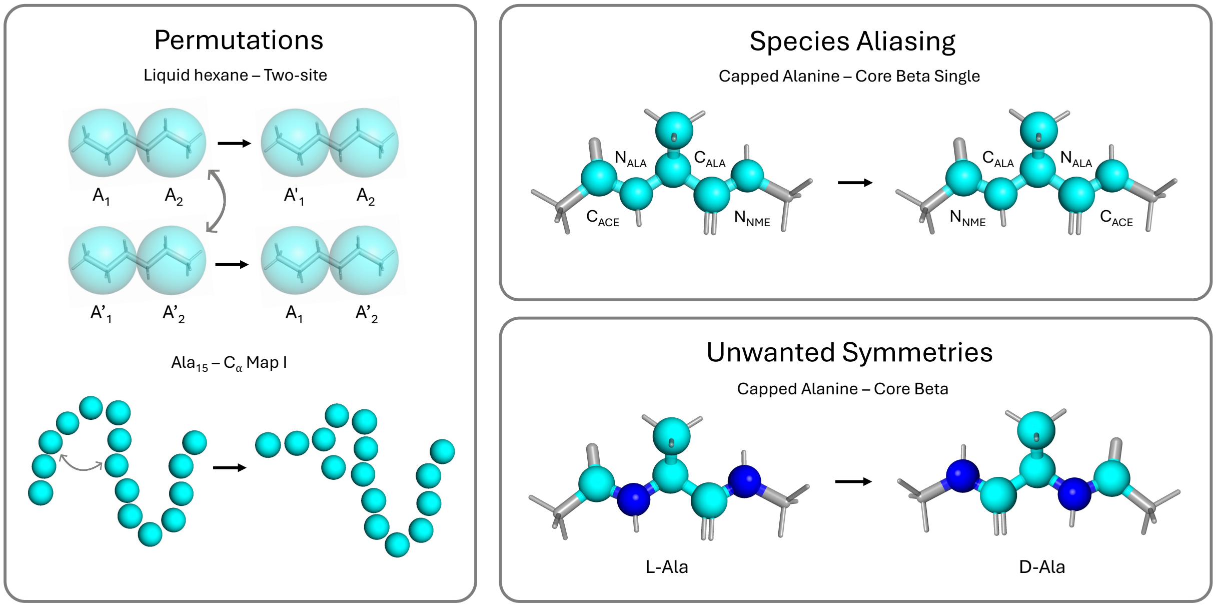

Figure S3: Overview of the different problems encountered when using equivariant machine learning potentials for coarse-grained potentials.

In this section, we give a brief explanation of the symmetries occurring in the capped amino acid systems. An overview can be seen in Figure S3. One symmetry was observed when using the Core Beta mapping with a single species, in which the dihedral FES is mirrored across the diagonal $\phi = -\psi$ (Figure S4a, column 2). Since MACE and other MLPs are permutation invariant, beads can effectively switch roles as long as the geometric environment is preserved. If one takes the perspective of the $C_\alpha - C_\beta$-axis, it is not hard to see how the front and back look very similar. During the simulation, the front and back are symmetrized and freely switch roles. This can also be clearly seen in the length distribution of the $N_{Ala} - C_\alpha$ and $C_\alpha - C_{Ala}$ bonds (Figure S4b). In the reference system, the $N_{Ala} - C_\alpha$ bond is slightly shorter as the $C_\alpha - C_{Ala}$ bond. However, since roles switch during the MLP simulation, the average is obtained. It is important to note, that at each point in time the front and back only occur *exactly* once. This means, that if one were to adjust the ordering when calculating the bonds/dihedrals, the correct FES would be obtained.

Another symmetry (Figure S4c) is obtained due to the enantiomerization of the capped

amino acids. As soon as one atom around the chiral center (here the C_α atom) is removed, the chiral inversion becomes accessible by inverting the improper dihedral. This inversion is coupled to a high-energy, planar transition state, making transitions rare events. If both two atoms around the chiral center are removed, the formal chirality is lost. Transitions between the two "enantiomers" now become simple, unhindered rotations of the backbone dihedrals ϕ/ψ.

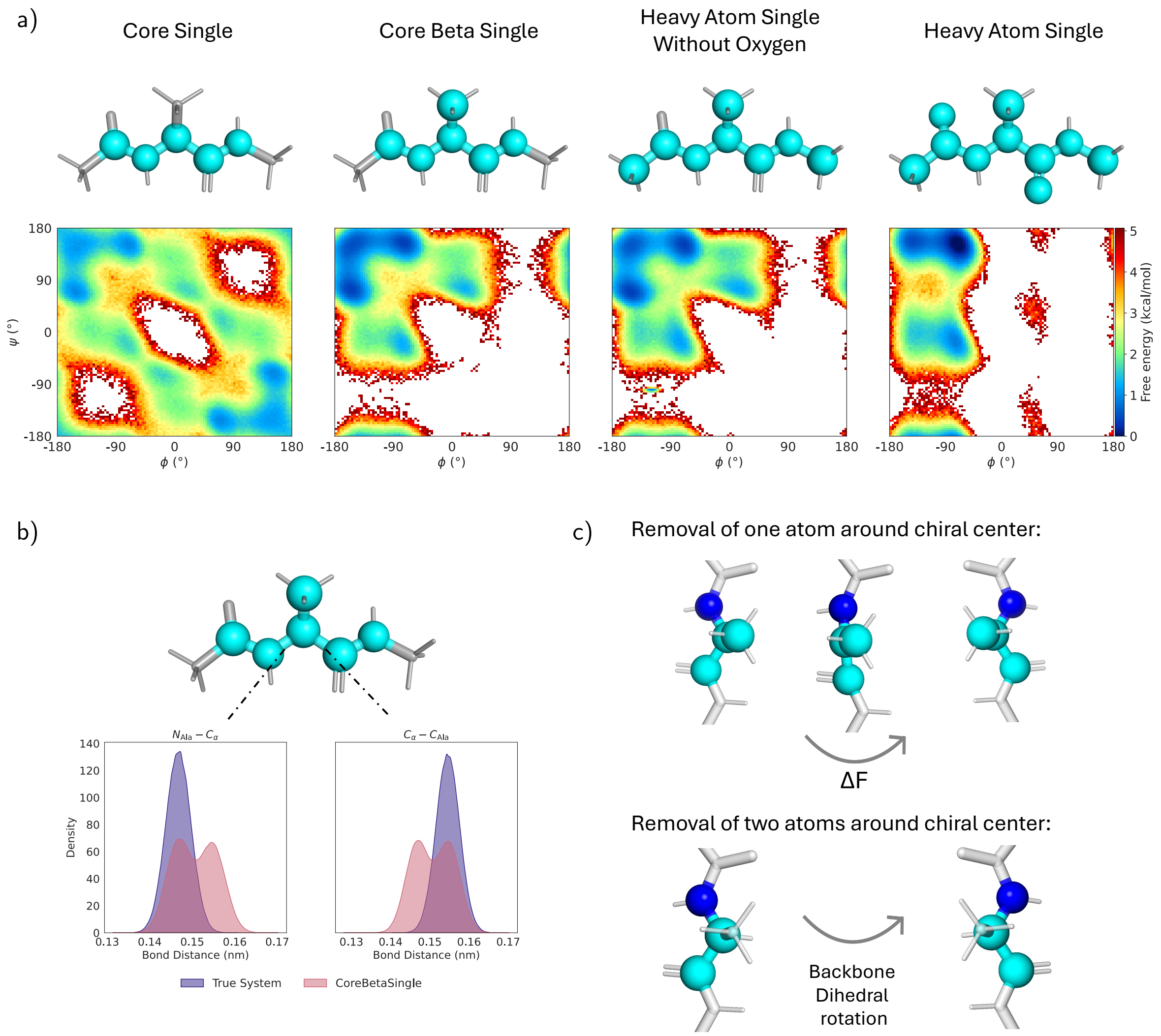

Figure S4: (a) Observed symmetries in different mappings capped alanine with a single species. (b) $N_{Ala} - C_\alpha$ and $C_\alpha - C_{Ala}$ bond lengths in the reference and Core Beta Single simulation.

Stability Analysis

Liquid Hexane

Analysis of Simulation Stability and k_BT Thresholds To quantify the stability of the coarse-grained machine learning potential (CG-MLP) simulations, a thermal energy threshold was implemented to detect the onset of numerical instabilities. We utilized a heuristic cutoff of 5 kJ/mol (approximately $2k_BT$) to identify catastrophic failures.

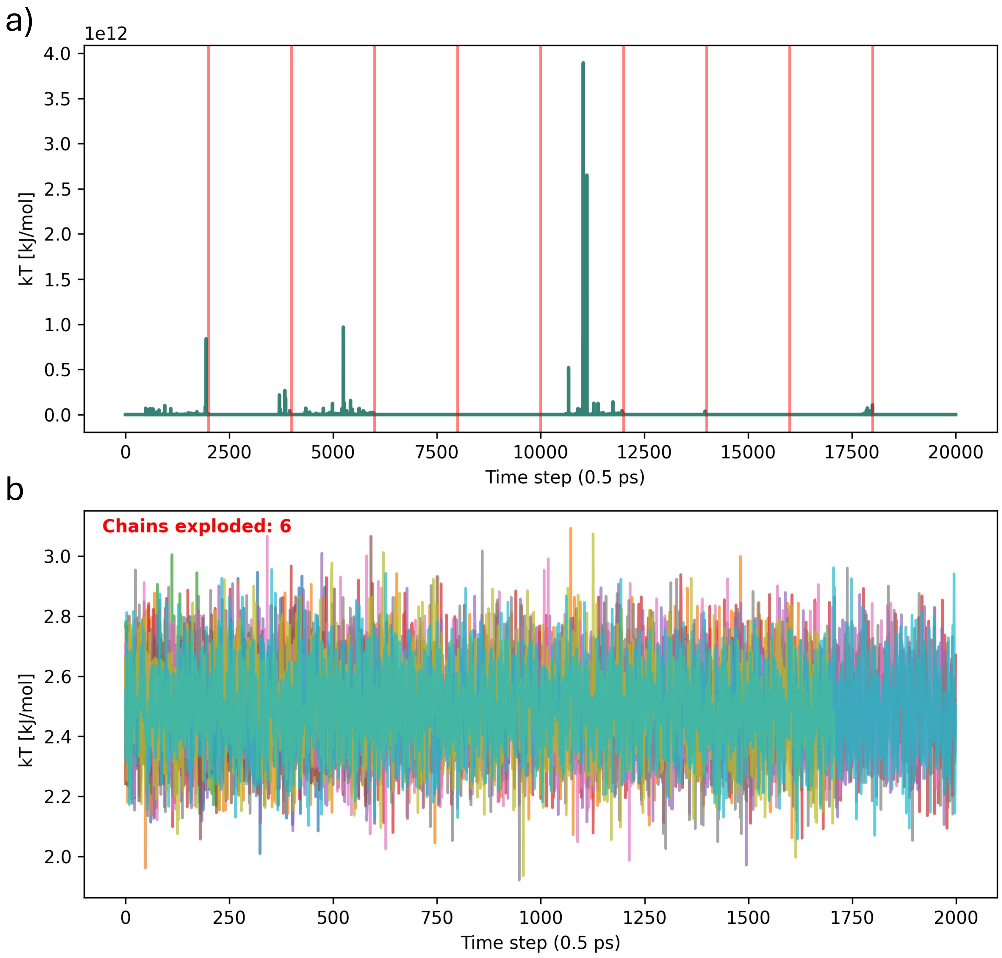

Figure S5: Detection of simulation instability. Example of observed k_BT for 10×1 ns liquid hexane CG-MLP ($N_{avg} \approx 20, L = 2, \nu = 3$) simulations. (a) Concatenated time series with red lines indicating the start of a new simulation. Exploded simulations are clearly visible as catastrophic failures. (b) Overlaid trajectories, in which simulations were terminated if k_BT exceeded 5 kJ/mol.

These failures are characterized by the MLP extrapolating into unsampled regions, leading to unphysical states, such as extreme bead overlaps that do not exist in the reference atomistic ensemble Consequently, the excluded high-energy frames represent numerical artifacts rather than legitimate high-energy Boltzmann states. Figure S5 illustrates the distinction between stable trajectories and those exhibiting numerical "explosions," where the energy diverges rapidly.

Sensitivity Analysis of Stability Thresholds We verified that the assessment of model stability is robust against the specific choice of the energy threshold by comparing the stability metrics using thresholds of of 3, 5, 10, and 20 kJ/mol. As shown in Table S7, the classification of stable trajectories and reported simulation lengths remain identical for all thresholds $\geq$ 5 kJ/mol. Table S6 summarizes the total number of unstable simulations across different model architectures using the standard 5 kJ/mol threshold. Additionally, we report the number of simulations that remained stable for the full 1 ns (Table S6), complementing Table 1 of the main text.

Table S6: **Number of unstable simulations for liquid hexane CG-MLP models.** Stability was assessed at a thermal energy threshold of 5 kJ/mol. All models were trained using the same random seed, and 50 simulations were performed for each configuration.

	$N_{avg} \approx 10$			$N_{avg} \approx 20$		
Model	$\boldsymbol{\nu = 1}$	$\boldsymbol{\nu = 2}$	$\boldsymbol{\nu = 3}$	$\boldsymbol{\nu = 1}$	$\boldsymbol{\nu = 2}$	$\boldsymbol{\nu = 3}$
Two-site	50	50	43	50	50	23
Three-site	50	2	8	50	25	9
Four-site	0	0	34	49	14	47

Table S7: **Stability comparison across varying thermal energy thresholds.** Results for the three-site model ($N_{avg} \approx 10, L = 2$). Stability is reported as mean length (ps); subscripts are standard deviations. Parentheses denote the number of stable trajectories out of 50.

Threshold	$\nu = 1$	$\nu = 2$	$\nu = 3$
3 kJ/mol	$931_{(220)}$ (45)	$374_{(297)}$ (2)	$429_{(348)}$ (8)
5 kJ/mol	$1000_{(0)}$ (50)	$379_{(294)}$ (2)	$446_{(350)}$ (8)
10 kJ/mol	$1000_{(0)}$ (50)	$379_{(294)}$ (2)	$446_{(350)}$ (8)
20 kJ/mol	$1000_{(0)}$ (50)	$379_{(294)}$ (2)	$446_{(350)}$ (8)

Amino Acids and Polyalanine

Next to the stability analysis of the liquid hexane simulations, here we report the stability of the capped amino acid and polyalanine simulations. In each case, the distance-based stability criterion of Fu et al.[8] was applied by checking whether the distance between the bonded atoms deviates by more than 0.05 nm from the equilibrium bond length. The equilibrium bond length is calculated as the mean of the reference simulation for all matching bond types and frames. Table S8 shows the mean and standard deviation of the length of the performed 100 × 5 ns NVT simulations that are the basis of the free energy surfaces in the main text.

For capped amino acid systems, we use this approach for backbone beads $C_{ACE}-N-C_\alpha-C-N_{NME}$, which are preserved in all tested mappings. For capped alanine, the equilibrium bond lengths of $C_{ACE}-N$, $N-C_\alpha$, $C_\alpha-C$ and $C-N_{NME}$ are 0.115, 0.127, 0.134 and 0.116 nm, respectively. In the case of the United Atom mapping, these move slightly, which is accounted for. For the polyalanine system, we filter based on the bond distance of adjacent C_α beads with an equilibrium bond length of 0.386 nm.

Capped Alanine NVE Simulations

For capped alanine, we also performed 100 × 100 ps NVE simulations and checked the number of chains that violated the distance-based stability criterion (Figure S6).

Table S8: Stability (ns) of different coarse-graining mappings, obtained from 100 independent 5-ns NVT simulations (atomistic: dt = 0.5 fs, all other: dt = 2 fs). Green cells mark the highest stability values.

**Map**	**Ala$_2$**	**Thr$_2$**	**Gly$_2$**	**Pro$_2$**	**Polyalanine**
Baselines					
Atomistic	5000.0 ± 0.0	–	–	–	–
Heavy Atom	5000.0 ± 0.0	–	–	–	–
United Atom	5000.0 ± 0.0	–	–	–	–
Core Maps					
Core	5000.0 ± 0.0	5000.0 ± 0.0	5000.0 ± 0.0	5000.0 ± 0.0	–
Core Map II	5000.0 ± 0.0	–	–	–	–
Core Single	5000.0 ± 0.0	5000.0 ± 0.0	4024.8 ± 1445.5	3988.8 ± 1595.4	–
Core β Maps					
Core Beta	5000.0 ± 0.0	5000.0 ± 0.0	–	5000.0 ± 0.0	–
Core Beta Map II	5000.0 ± 0.0	–	–	–	–
Core Beta Single	5000.0 ± 0.0	5000.0 ± 0.0	–	5000.0 ± 0.0	3763.5 ± 1674.4
CA Maps					
CA Map I	–	–	–	–	177.6 ± 343.9
CA Map II	–	–	–	–	1442.7 ± 1444.0
CA Map III	–	–	–	–	528.3 ± 627.3
CA Map IV	–	–	–	–	84.1 ± 127.7

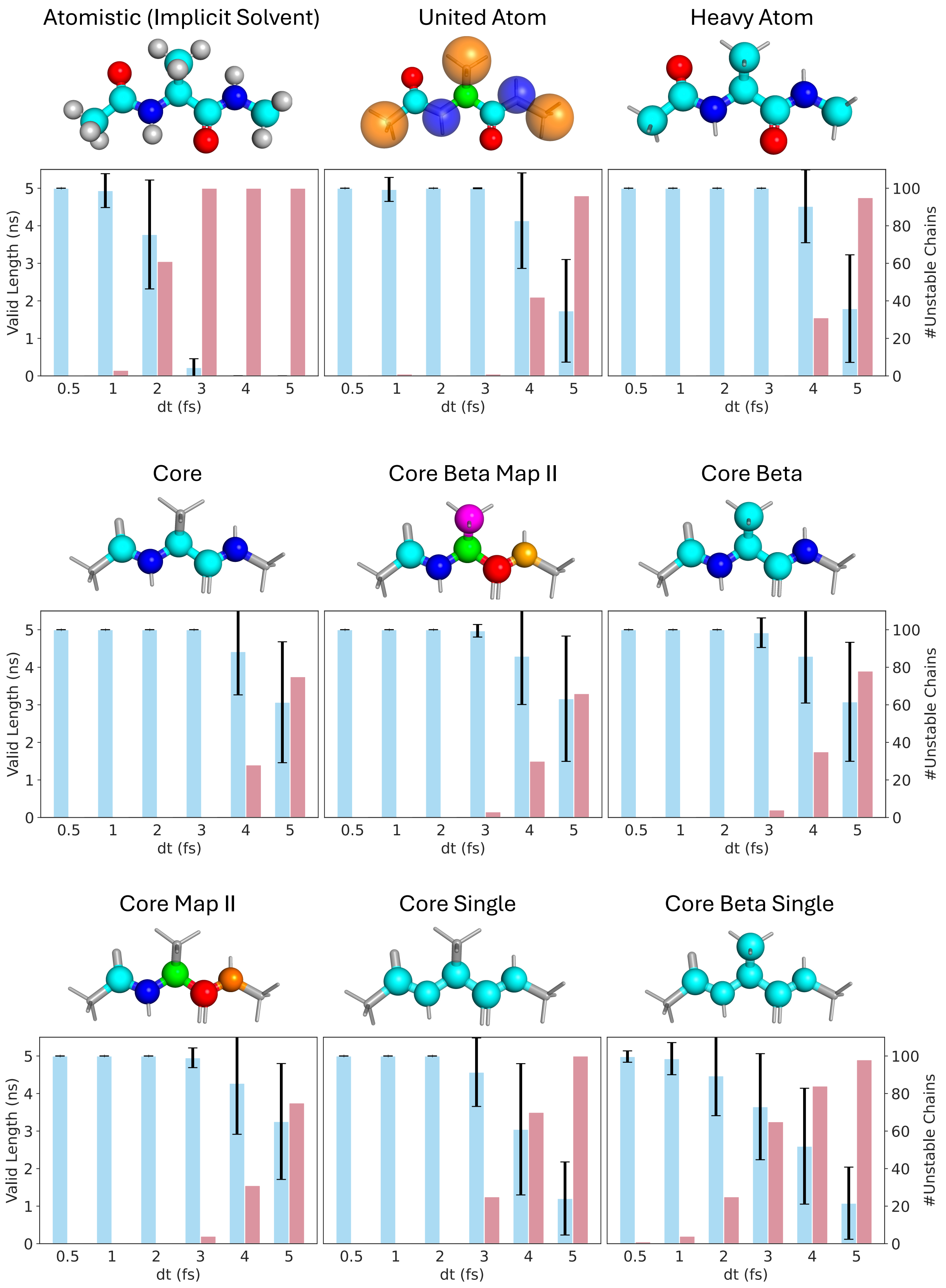

Figure S6: Results of the NVE timestep analysis for different CG mappings of capped alanine based on 100 × 100 ps simulations. Error bars show one standard deviation.

Structural Analysis

Liquid Hexane

In Figure S7 and S8 the full structural analysis of the four- and three-site liquid hexane model can be found.

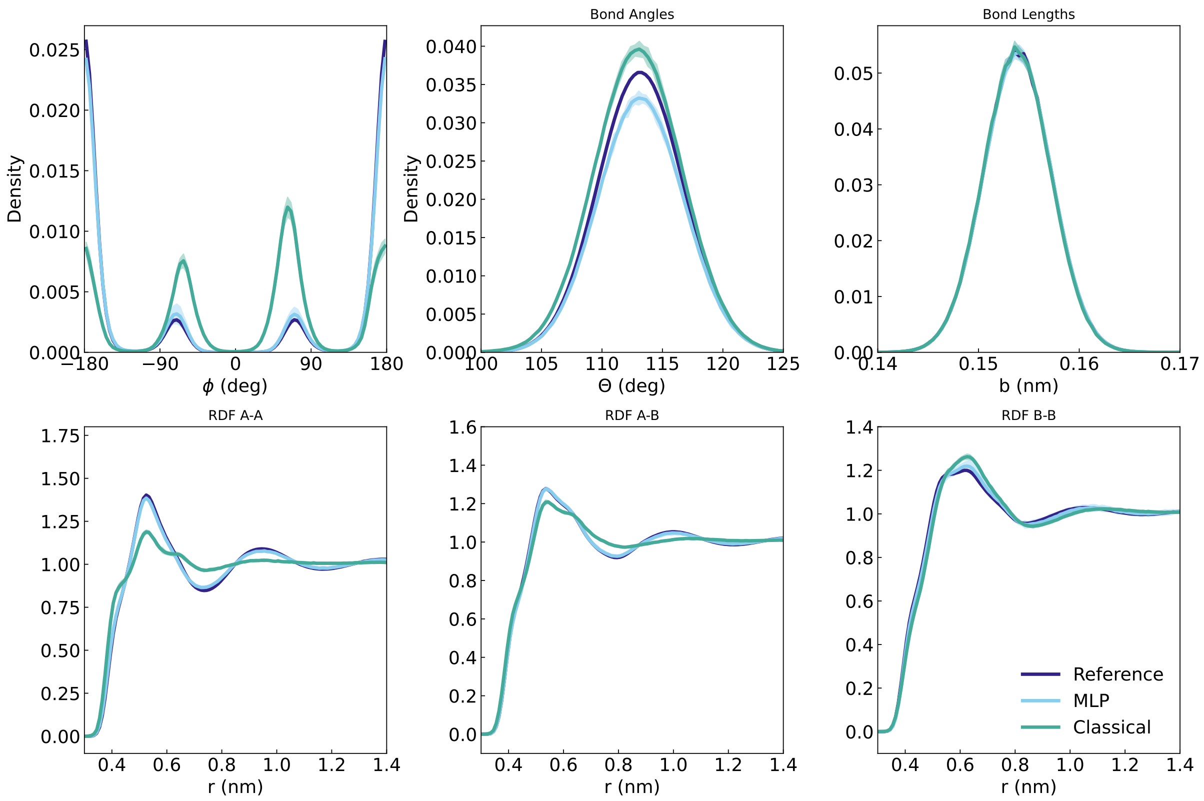

Figure S7: Full Results of the MLP ($\nu = 2$, $L = 2$, $N_{avg} \approx 20$) and classical potential for the four-site coarse-grained representations of hexane. Results show the mean $\pm$ 3 standard deviations of 10 $\times$ 1000 ps simulations. First row shows bonded population density metrics: Dihedral, Angle, Bond population. The second row shows the radial distribution function (RDF) of A-A, A-B and B-B beads.

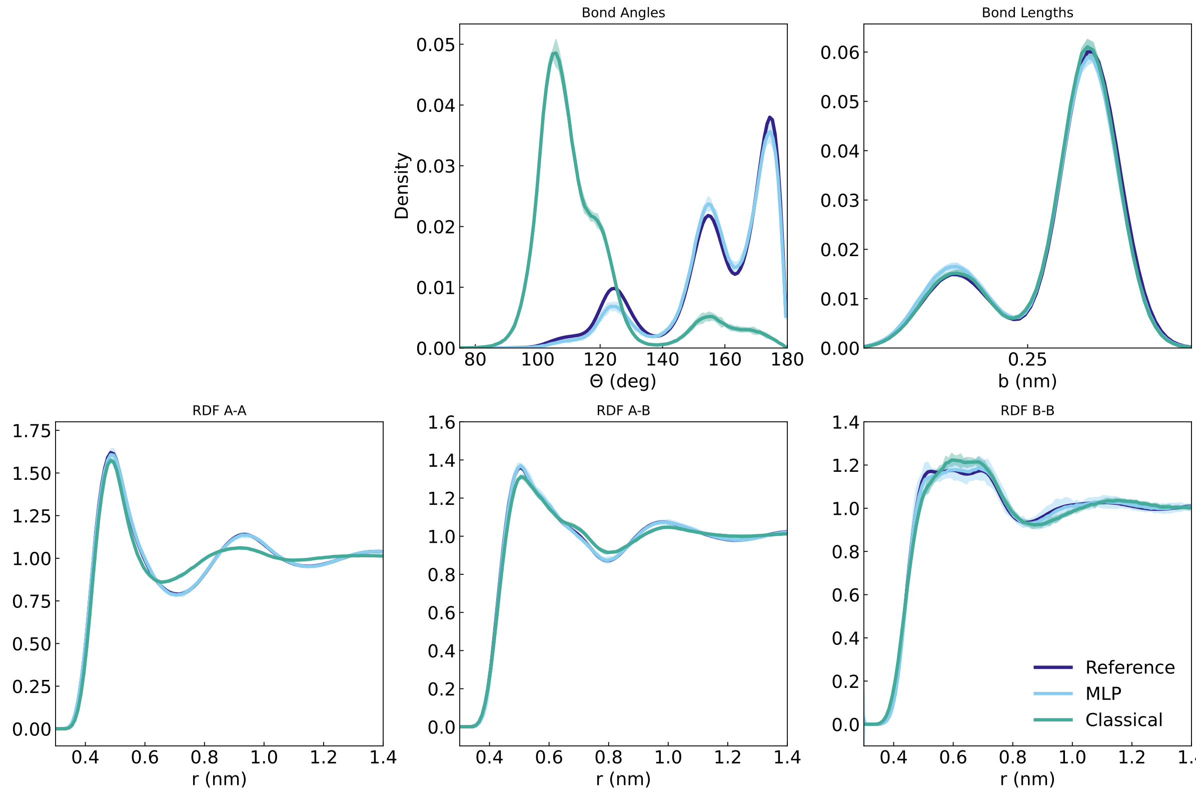

Figure S8: Full Results of the MLP ($\nu = 2$, $L = 2$, $N_{avg} \approx 20$) and classical potential for the three-site coarse-grained representations of hexane. Results show the mean $\pm$ 3 standard deviations of 10×1000 ps simulations. First row shows bonded population density metrics: Angle and Bond population. The second row shows the radial distribution function (RDF) of A-A, A-B and B-B beads.

Angular Distribution Function To characterize the local structural orientation and many-body correlations in the liquid hexane model, we utilize angular distribution functions (ADFs). The ADF $P(\theta)$ represents the probability density of finding a specific angle θ formed by three particles. In a coarse-grained context, this describes the distribution of angles between bonded or nearest-neighbor beads:

$$P(\theta) = \frac{1}{N_{\text{triplets}}} \left\langle \sum_{i \neq j \neq k} \delta(\theta - \theta_{ijk}) \right\rangle \tag{2}$$

where θ_{ijk} is the angle formed by the vectors $\vec{r}_{ji}$ and $\vec{r}_{jk}$.

We evaluated the ADF for the different liquid hexane mappings within 0.8 nm, defined

by the first minimum of the radial distribution function (first solvation shell). The MLP demonstrates an excellent fit to the reference data, whereas the classical CG model fails to accurately capture the AAA-type angular distributions in the three- and four-site mappings (Figure S9). This discrepancy highlights the ability of the MLP to resolve complex many-body correlations that the fixed functional forms of the classical CG potentials fail to capture.

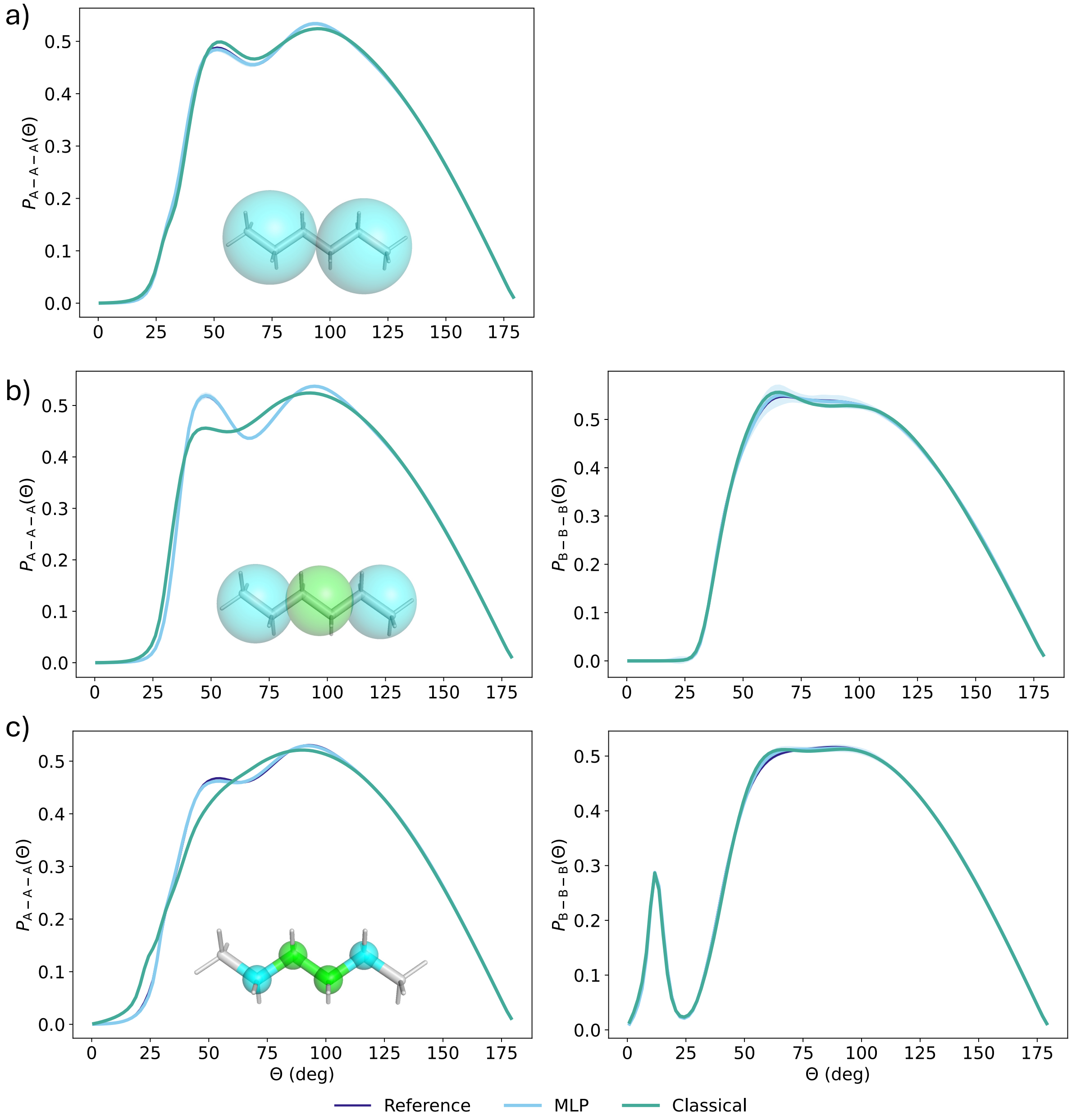

Figure S9: Angular Distribution Function for liquid hexane models. Results show the mean $\pm$ 3 standard deviations of 10×1000 ps simulations for the MLP ($\nu = 2$, $L = 2$, $N_{avg} \approx 20$) and classical potential. (a) A-type ADF for the two-site mapping. (b) A- and B-type ADF for the three-site mapping. (c) A- and B-type ADF for the four-site mapping.

Bond Swaps in Heavy Model To determine if the unphysical bond-switching behavior observed in the two-site hexane model originated from limited model expressivity, we trained a higher-capacity MACE architecture with increased depth ($L = 3$) and correlation order

($\nu = 2$). While this more complex model delayed the onset of instability, extending the simulation to 10 ns revealed that unphysical bond permutations still accumulate, eventually affecting all beads in the system as shown in Figure S10. We found that further increasing the capacity (e.g., $\nu = 4$) was computationally prohibitive, exceeding the memory limits of an 80 GB NVIDIA A100 GPU for even small systems. These results indicate that bond swaps are not a consequence of model capacity, but rather a fundamental limitation of the two-site mapping resolution. In this representation, the overlap between bonded and non-bonded length scales creates an inherent topological ambiguity that even highly expressive potentials cannot fully resolve.

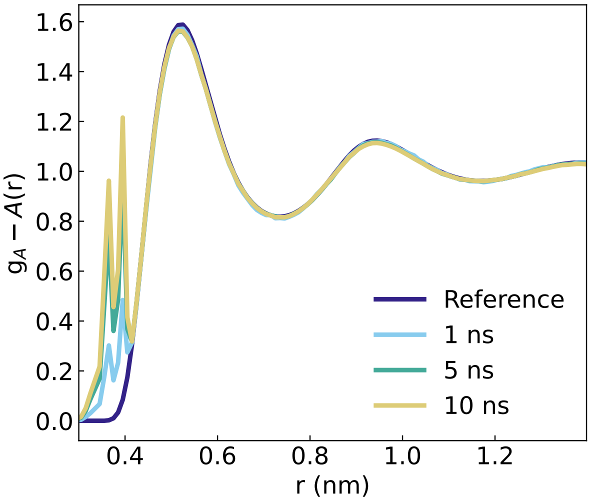

Figure S10: Radial Distribution Function (RDF) of the liquid hexane two-site model using a higher-capacity architecture ($L = 3, \nu = 2$). While the artifact at ≈ 0.35 nm is small at 1 ns, it grows significantly over 10 ns, indicating that increased capacity only delays the onset of unphysical bond swapping.

Capped Amino Acids

In addition to capped alanine, we also tested the low-resolution backbone mappings on three other amino acids: glycine, threonine, and proline. The resulting free energy surfaces of the backbone dihedrals can be seen in Figure S11.

Overall, the observed symmetries are the same as those observed for capped alanine.

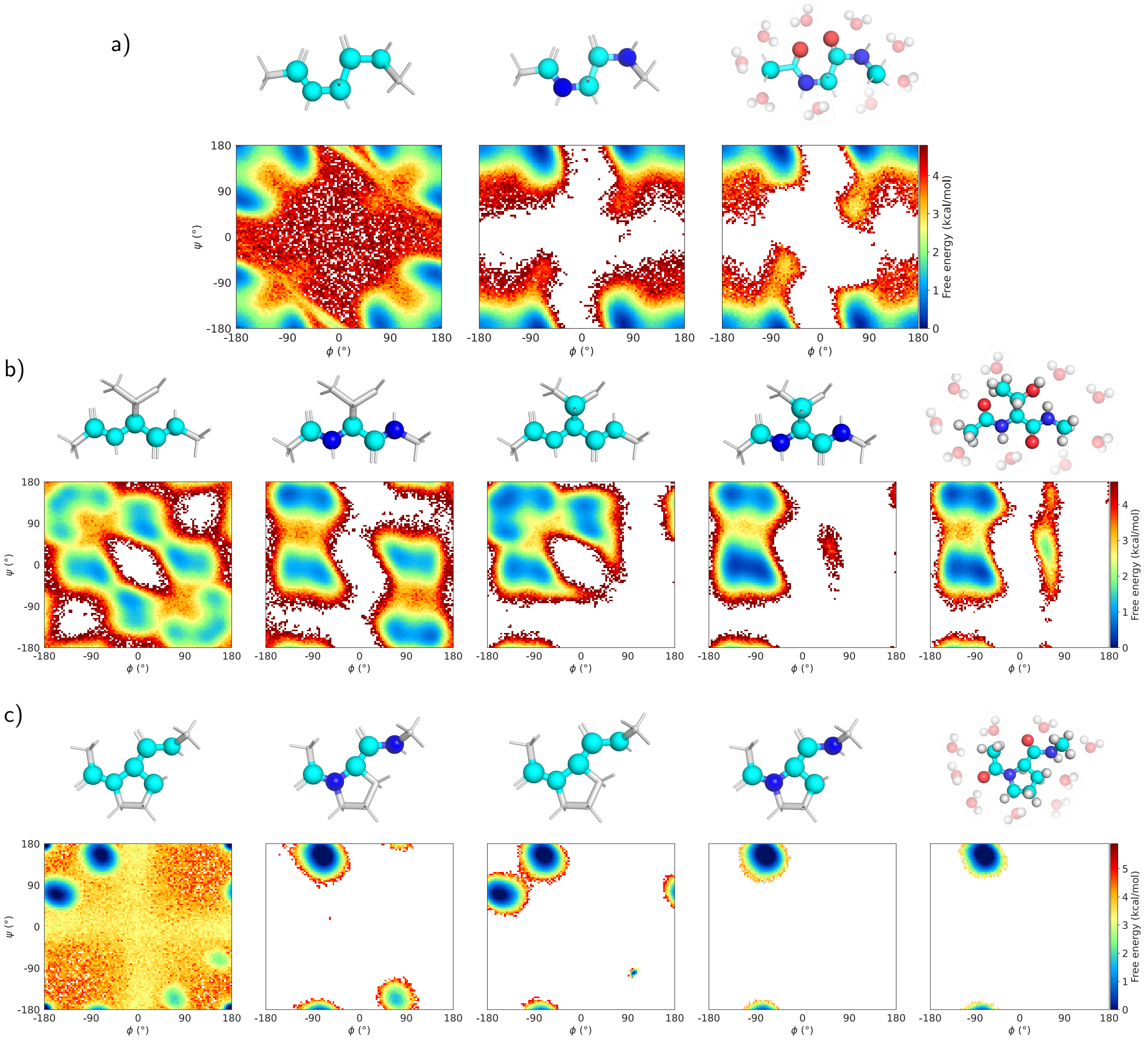

Figure S11: Ramachandran plots for backbone dihedrals based on trained MACE potentials for different CG mappings for (a) glycine (b) threonine and (c) proline. The first plot of each row shows the reference explicit solvent simulation on which models where trained.

Because glycine does not contain a side chain and is therefore not chiral, only the Core mappings were tested. For all amino acids, the Core/Core Beta mapping with a single species again fails to distinguish the two dihedrals and adds spurious samples across $\phi = -\psi$. Removing the side-chain C_β bead in the case of threonine and proline again causes a $\pm$ symmetry, or enantiomer switch, for both dihedrals.

Chiral Inversion

Metadynamics Simulations We apply well-tempered metadynamics along the improper dihedral angle centered around the C_α bead, which includes the C_β-bead, as well as the carbon and nitrogen of the backbone. Biased simulations of the CG systems were run for 5 ns with a 1 fs time step, while the atomistic (implicit solvent) model was simulated for 2.5 ns with a 0.5 fs time step. We add Gaussians every 500 steps with a height of 0.2 and a standard deviation of 0.1 and a bias factor γ of 5. We show the convergence of the metadynamics runs in Figure S12a.

Wait Time Estimation The mean wait time τ for the chiral inversion event was estimated using Transition State Theory (TST). The rate constant k for the transition is defined by the Eyring equation:

$$k = \frac{k_B T}{h} \exp\left(-\frac{\Delta F^\ddagger}{k_B T}\right) \tag{3}$$

where k_B is the Boltzmann constant, h is Planck's constant, T is the temperature (300 K), and $\Delta F^\ddagger$ is the free energy barrier height obtained from the metadynamics simulations. The mean wait time is then calculated as the inverse of the rate constant $\tau = \frac{1}{k}$.

This approach assumes a transmission coefficient (κ) of unity. This assumes that every trajectory that reaches the transition state proceeds to the other enantiomer without recrossing.[9] Although κ is often less than unity in condensed phases due to solvent friction and barrier recrossing events, computing the exact dynamic factor requires extensive unbiased trajecto-

ries that are computationally inaccessible for these barrier heights. Thus, the TST estimate provides a theoretical lower bound for the wait times determined in Figure S12b.

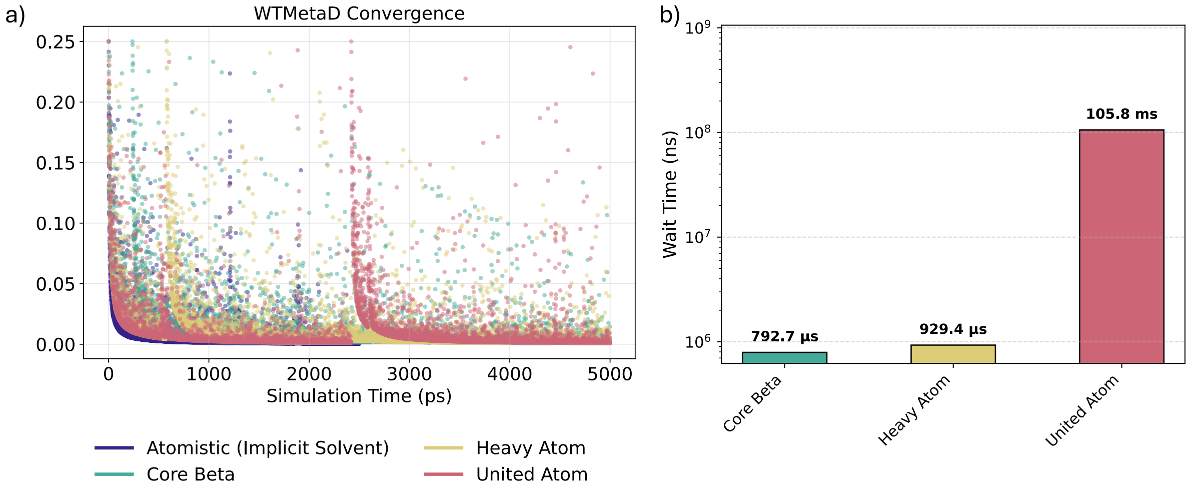

Figure S12: (a) Convergence of the WTMetaD simulations. (b) Estimated mean wait times for chiral inversion at 300 K calculated from the respective free energy barriers using transition state theory.

Polyalanine

In addition to a C_α-mapping with unique species (Map II) for each bead, we also tested a symmetric (Map III), single (Map I) and alternating species (Map IV) encoding (Figure S13). To all simulations, the distance-based filter criterion was applied (see Methods). All C_α-based mappings fail to reproduce the correct helix formation. They are also notoriously unstable (Table S8); in the case of the alternating and single species encoding this is also a product of bond permutations - similar to the ones observed the two-site liquid hexane model.

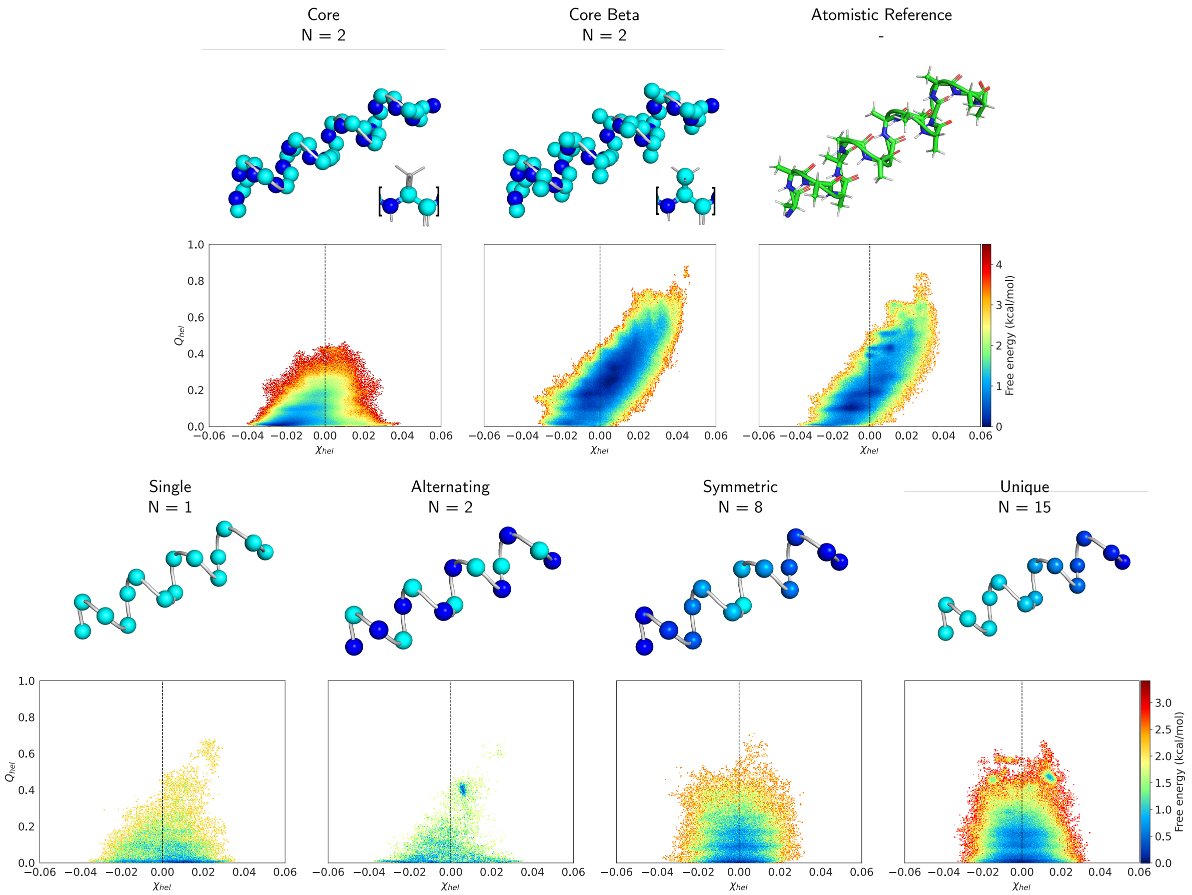

Figure S13: Helicity and handedness of 100 × 5 ns polyalanine trajectories based on different C_α encoding. For each mapping, we show the number of species N used, a visual representation of the full helix, as well as the 2D helicity formation plot.

References

(1) Schrödinger, L.; DeLano, W. PyMOL. `http://www.pymol.org/pymol`.

(2) Rühle, V.; Junghans, C. Hybrid approaches to coarse-graining using the VOTCA package: liquid hexane. *Macromolecular Theory and Simulations* **2011**, *20*, 472–477.

(3) Schoenholz, S. S.; Cubuk, E. D. JAX, MD A framework for differentiable physics. *Journal of Statistical Mechanics: Theory and Experiment* **2021**, *2021*, 124016.

(4) Fuchs, P.; Thaler, S.; Röcken, S.; Zavadlav, J. chemtrain: Learning deep potential models

via automatic differentiation and statistical physics. *Computer Physics Communications* **2025**, *310*, 109512.

(5) Ruhle, V.; Junghans, C.; Lukyanov, A.; Kremer, K.; Andrienko, D. Versatile object-oriented toolkit for coarse-graining applications. *Journal of chemical theory and computation* **2009**, *5*, 3211–3223.

(6) Abraham, M. J.; Murtola, T.; Schulz, R.; Páll, S.; Smith, J. C.; Hess, B.; Lindahl, E. GROMACS: High performance molecular simulations through multi-level parallelism from laptops to supercomputers. *SoftwareX* **2015**, *1*, 19–25.

(7) Batzner, S.; Musaelian, A.; Sun, L.; Geiger, M.; Mailoa, J. P.; Kornbluth, M.; Molinari, N.; Smidt, T. E.; Kozinsky, B. E (3)-equivariant graph neural networks for data-efficient and accurate interatomic potentials. *Nature communications* **2022**, *13*, 2453.

(8) Fu, X.; Wu, Z.; Wang, W.; Xie, T.; Keten, S.; Gomez-Bombarelli, R.; Jaakkola, T. Forces are not enough: Benchmark and critical evaluation for machine learning force fields with molecular simulations. arXiv (accessed 2026-01-20), DOI: 10.48550/arXiv.2210.07237.

(9) Hänggi, P.; Talkner, P.; Borkovec, M. Reaction-rate theory: fifty years after Kramers. *Reviews of modern physics* **1990**, *62*, 251.
